# Investigation of Thermodynamic, Kinetic, and Isothermal Parameters for the Selective Adsorption of Bisphenol A

**DOI:** 10.1021/acsomega.2c01975

**Published:** 2022-05-24

**Authors:** Recep Üzek, Serap Şenel, Adil Denizli

**Affiliations:** Department of Chemistry, Faculty of Science, Hacettepe University, 06800 Ankara, Turkey

## Abstract

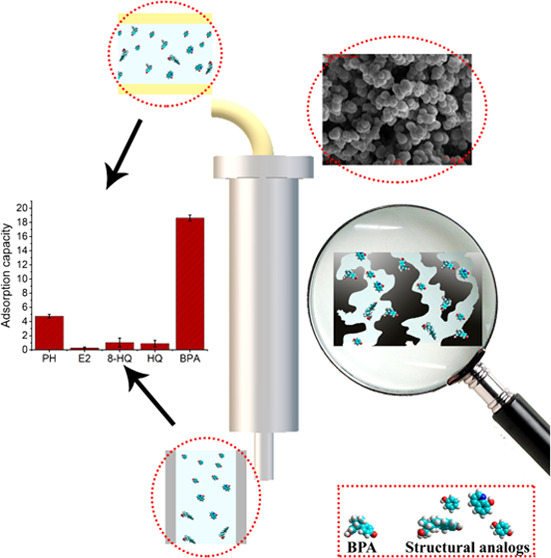

Herein, a novel imprinted solid-phase extraction cartridge was fabricated to investigate the kinetic, thermodynamic, and isothermal parameters for the selective adsorption of Bisphenol A (BPA). The imprinted polymeric cartridges (BMC) for the BPA adsorption were fabricated in the presence of a template and functional monomer using the in situ polymerization technique. To prove the efficiency and selectivity of BMC, the nonimprinted polymeric cartridges (BNC) and the empty polymeric cartridges (EC) were also fabricated with and without functional monomer using the same manner for the preparation of BMC. The characterization of cartridges was performed by elemental analysis, Fourier transform infrared (FTIR) spectroscopy, scanning electron microscopy (SEM), Brunauer–Emmett–Teller (BET) surface area measurements, and swelling tests. BPA removal studies were performed by analyzing some parameters such as temperature, BPA concentration, flow rate, salt type, and concentration. The highest capacity was determined as 103.2 mg BPA/g polymer for a 0.75 mL/min flow rate of 0.75 M (NH_4_)_2_SO_4_ containing 200 mg/L BPA solution at 50 °C. NaOH (1.0 M) was used as a desorption agent. The reusability performance was examined by performing 10 consecutive cycles. The solid-phase extraction (SPE) performance was also checked to determine the enrichment and extraction recovery factors for tap water and synthetic wastewater samples. Temkin, Langmuir, Freundlich, and Dubinin–Radushkevich isotherm models were applied to BPA adsorption data examining the adsorption mechanism, surface properties, and adsorption degree. The most suitable isotherm model for BPA adsorption was determined as the Langmuir isotherm model. The thermodynamic parameters (Δ*G*°, Δ*H*°, and Δ*S*°) were investigated to reveal the thermodynamics of adsorption. Adsorption thermodynamic parameters (Δ*H*°, Δ*S*°, and Δ*G*°) were calculated using the thermodynamic equilibrium constant (thermodynamic equilibrium constant, *K*°) values that change with temperature. It was determined that BPA adsorption was spontaneous (Δ*G*° < 0) and endothermic (Δ*H*° > 0) and entropy increased (Δ*S*° > 0) at the temperatures studied in the BPA adsorption process. The rate control step in the adsorption process was examined by applying pseudo-first-order and pseudo-second-order kinetic models to the adsorption data for the investigations of BPA adsorption kinetics, and the pseudo-second-order kinetic model was found to be more suitable for describing BPA adsorption kinetics. In examining the selectivity of cartridges, structural analogues of hydroquinone, phenol, β-estradiol, and 8-hydroxyquinoline were tested.

## Introduction

**Figure 1:**
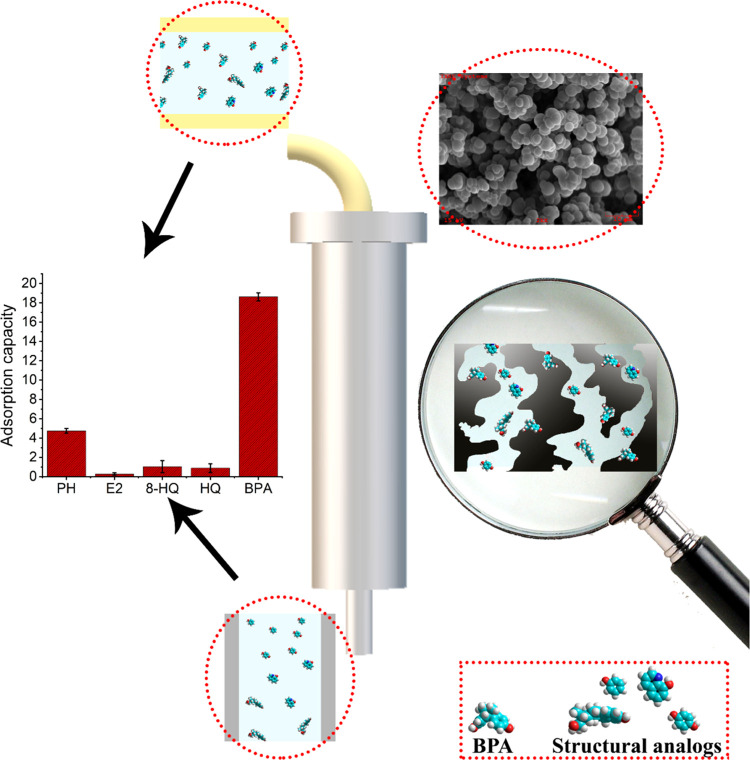
Illustration of extraction mechanism of imprinted cartridges for BPA removal.

Bisphenol A (BPA) is the main ingredient utilized in the fabrication of polycarbonate and epoxy resin manufacture. So, it participates in many consumer and industrial products.^1,2^ BPA can enter water sources via municipal wastewater and industrial discharge. Food, soil, and air are other sources of exposure.^1,3^ Health problems including reproductive abnormalities, breast, and prostate cancer have been associated with low-dose (ng/L) exposure to BPA.^4−8^ It disrupts the normal functioning of the endocrine system. Therefore, its precise determination and removal from aquatic media become highly important due to health considerations. Considering the concentration of BPA in aquatic media, it becomes necessary to preconcentrate by applying the extraction process before the detection of BPA. Three extraction techniques (liquid–liquid (LLE), solid-phase (SPE), and micro-solid-phase (SPME)) have been mostly preferred for the isolation and preconcentration of BPA.^9^ High-performance liquid chromatography (HPLC), gas chromatography/mass spectrometry (GC/MS), or electrochemical methods can be preferred for the analysis of extracts.^10−12^ In the extraction process, adsorbent materials such as clay minerals, nanoparticles, carbon-based materials, and molecularly imprinted materials were used to preconcentrate and remove BPA from the aqueous solution.^13−21^ Molecularly imprinted polymers (MIPs) have great potential as SPE sorbents due to the advantages of thermal and chemical stabilities, low cost, and ease of synthesis.^22−24^ Chemical association or physical interaction of template with functional monomer, polymerization including initiator, porogen, crosslinker, and template removal are the main steps in the synthesis of MIPs.^25,26^ The resulting MIP adsorbents with three-dimensional (3D) recognition sites for the template are used for the selective extraction of the template. MIPs can be synthesized in different forms such as nanoparticles, microparticles, in situ prepared monoliths, molecularly imprinted films, and membranes, considering the final approach.^26−31^ Monolithic MIPs have been extensively chosen in SPE applications because of a simple, one-step, in situ polymerization process to use directly as an SPE cartridge without any necessity of grinding, sieving, and packing.^31^ Moreover, the template amounts required in the preparation of monolithic MIPs are much lower than that in other methods. The monolithic MIPs have good permeability and high surface area, improving separation with higher performance due to their greater porosity.^32,33^ Ren et al. used the sol–gel process to deposit diethylenetriamine pentaacetic acid functional monomer and tetraethylorthosilicate crosslinker on silica nanoparticles. The adsorption amount of the resulting BPA-MIP was 30.26 μmol BPA/g.^34^ The noncovalent mode was applied for BPA-imprinted polymer using MAA and 2-vinyl pyridine (2-VP) as monomers. The low detection limit (0.2 ng/g), good stability, and linearity were the advantages.^35^ MIP prepared with 4-vinyl pyridine (VP) as a functional monomer was tested to extract BPA from various environmental and biological samples and reported as an effective MISPE sorbent in the range of 2–20 μM.^36^ The precipitation polymerization method was used for BPA-MIPs using the same functional monomer, and the detection limits varying between 0.1 and 3.8 ng/g were achieved in commercial honey samples.^37^ Sasaki et al. show that ATRP-based BPA-MIPs had higher selectivity than BPA-MIPs synthesized by radical polymerization.^38^ Herein, the molecularly imprinted polymeric cartridges were fabricated using amino acid functional monomer, *N*-methacryloyl-l-phenylalanine (MAPA), to selectively remove and preconcentrate BPA from an aqueous solution. Following characterization, the BPA extraction performance of the cartridges from an aqueous solution was studied. The extraction mechanism of imprinted cartridges for BPA removal is shown in Figure 1.

## Experimental Section

### Materials and Apparatus

Bisphenol A (BPA), ethylene glycol dimethacrylate (EDMA), azobisisobutyronitrile (AIBN), phenol, β-estradiol, hydroquinone, and 8-hydroxyquinoline were purchased from Sigma (St. Louis). All chemicals were of analytical purity grade (>97%) unless otherwise indicated. *N*-methacryloyl-l-phenylalanine (MAPA, *d* = 1.10 g/mL in ethanol) was obtained from NANOREG (Ankara, Turkey) company. Other used chemicals were of analytical purity grade (97%) obtained from Sigma (St. Louis). Ultrapure water (>18.2 MW cm^–1^) obtained using the ROpure LP device from Barnstead (Dubuque, IA) was utilized to prepare all aqueous solutions.

Polymeric cartridges were synthesized in 5 mL volume plastic injectors (diameter, 1.80 cm; height 5 cm). The Julabo brand (Variomag EC, Germany) water bath was used to prepare polymeric cartridges. The pH meter of Mettler Toledo (Schwerzenbach, Switzerland) was utilized to measure the solution pH. In BPA adsorption studies from an aqueous solution, the Watson–Marlow multichannel peristaltic pump (Wilmington, MA) was used.

Precisa XB220A (4 digits, Moosmattstrasse, Switzerland) precision scales were used in all weightings. Fourier transform infrared spectroscopy–attenuated total reflection (FTIR-ATR) and elemental analyzer (Thermo Scientific, Flash 2000-CHNS) were used for the structural characterization. In the surface area measurements of polymeric cartridges, an automatic surface area and pore size analyzer (Quantachrome NOVA 2000) was used to determine the surface morphology of scanning electron microscopy (SEM) (JEOL, JEM 1200EX, Tokyo, Japan). CH_3_COONa–CH_3_COOH, K_2_HPO_4_–KH_2_PO_4_, and Na_2_CO_3_–NaHCO_3_ pairs were used to prepare the buffer solutions at a concentration of 0.10 M. BPA concentrations in solutions were determined by the absorbance measurements taken with an ultraviolet (UV)–visible spectrophotometer (UV mini-1240, Shimadzu, Tokyo, Japan).

### Synthesis of the Polymeric Cartridges

The template–functional monomer ratio (precomplex) is highly important for the efficiency of imprinting and increasing selectivity. Therefore, the ratio was determined first (Table S1 and Figure S1, Supporting Materials). After determining the ratio, the polymeric cartridges were fabricated using in situ polymerization. The substances with the amounts used for the polymeric cartridges fabrication are given in Table S2 (Supporting Materials). The BPA-imprinted polymeric cartridges (BMC) are prepared as follows: the precomplex was formed by dissolving 90 mg of BPA in 1.10 mL of MAPA. Then, the precomplex, EDMA, and porogen (ethyl alcohol, EtOH) are mixed and stirred magnetically for 15 min. Azobisisobutyronitrile (AIBN) was added as an initiator to the solution. The final solution was filled into a 5 mL syringe and the polymerization was carried out in a water bath for 3 h at 70 °C (Figure S2). The nonimprinted polymeric cartridges (BNC) were produced by performing the same polymerization process without adding BPA to the polymerization solution. The polymeric cartridges (EC) were prepared without adding the precomplex to the monomer solution in the same polymerization process to compare the interaction of the functional monomer with the template molecule. The polymeric cartridges were washed three times with 50 mL of ethyl alcohol–ultrapure water (v/v, 50:50), and a 1.0 M NaOH solution (100 mL) was used to remove the template molecule. Finally, it was washed with ultrapure water and stored in a refrigerator at 4 °C until used in adsorption experiments.

### Characterization

The structural characterization was carried out with FTIR-ATR and the elemental analyzer. FTIR-ATR analyses of polymeric cartridges were performed by measuring the total amount of reflection on the surface in the 400–4000 cm^–1^ wave count range. The MAPA amounts involved in the structure of BMC and BNC were determined by the elemental analyzer. For elemental analysis, 1.0 mg of sample was weighed with a sensitivity of ±0.0001 g and placed in the device chamber, and after burning, the amounts of carbon, hydrogen, nitrogen, and sulfur in the sample were determined.

The equilibrium swelling analyses of the polymeric cartridges were performed using ultrapure water as follows; the dried polymer was weighed with a sensitivity of ±0.0001 g and thrown into 50 mL of ultrapure water. After some time at a constant temperature (25 ± 0.5 °C), it was removed from the water and weighed (0.0001 g sensitivity) by removing water from the surface with filter paper. Dry weights and wet weights were determined, and the degree of swelling and porosity were determined using the following equations
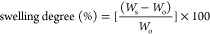

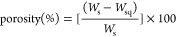
where *W*_o_ and *W*_s_ refer to the masses of the dry polymer and the swollen polymer, respectively, and *W*_sq_ refers to the mass of the wet polymer obtained by compressing the swollen polymer.

The surface characterizations were carried out using an auto surface area and pore size analyzer. Nitrogen was used as the adsorbed gas in the measurements (purity 99.9%), and the nitrogen source was liquid nitrogen and the temperature was 77 K. The gas removal from the sample was carried out in a vacuum at 184 °C for 5 h. Multipoint Brunauer–Emmett–Teller (BET) analysis was utilized to determine the pore sizes and the specific surface areas.

The surface and bulk structures were investigated by scanning electron microscopy (SEM) analysis. The surface of polymeric cartridges dried in vacuum was coated with gold in the vacuum, and SEM images were taken at different magnification rates.

### Adsorption Studies

The BPA adsorption performance of the polymeric cartridges was examined in a continuous system. The polymeric cartridge synthesized in the 5 mL injector was connected to the speed-adjustable peristaltic pump (Figure S3). BPA solutions at different concentrations were passed through the column while being stirred magnetically (150 rpm). To determine the optimum conditions for BPA adsorption, the effects of parameters such as pH (4.0–9.0), flow rate (0.25–2.0 mL/min), BPA initial concentration (25.0–300.0 mg/L), temperature (4–45 °C), salt type, and concentration on the adsorption capacity were examined. To keep the pH of the medium constant, the buffer solutions (acetate, phosphate, and carbonate) were used at 100 mM concentrations. For the effect of salt type and concentration, aqueous solutions of sodium sulfate, ammonium sulfate, and sodium chloride salts in concentrations of 0.1–1.0 M were prepared. The BPA concentrations were calculated using absorbance values measured at 280 nm. The following equation was used to calculate the adsorption capacity (*q*)
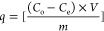
BPA concentrations before and after BPA adsorption are described by *C*_o_ and *C*_e_, mg/L, respectively. The mass of the polymeric cartridge and the volume of the BPA solution are defined by *m* (g) and *V* (L), respectively.

Hydroquinone (HQ), phenol (PH), β-estradiol (E2), and 8-hydroxyquinoline (8-HQ) were used as competitor substances in the selectivity studies to determine the sensitivity and selectivity of BMC. The chemical structures of BPA and competitor substances are given in Figure S4. Solutions of competitor substances (100 mL, 50.0 mg/L) prepared in pH 5 buffer were passed at a 0.75 mL/min flow rate through polymeric cartridges at 25 °C. The competitor concentrations in solutions were determined by taking absorbance measurements at the wavelength at which the UV–visible spectrophotometer showed maximum absorbance. The following equations are used to calculate the distribution coefficient (*k*_D_) and selectivity parameters (selectivity constant (*k*) and relative selectivity coefficient (*k*′)) of BMC and BNC:
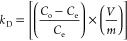
where *C*_o_ and *C*_e_ (mg/L) refer to the BPA concentrations before and after the BPA adsorption, respectively. The mass of the polymeric cartridge and the volume of BPA solution are denoted by *m* (g) and *V* (L), respectively.
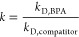

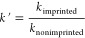
The desorption solution (1.0 M NaOH) was passed for 1 h at a 0.75 mL/min flow rate. Then, ultrapure water was used for washing to make it ready for use again. The cycles including adsorption–desorption–regeneration were repeated 10 times to determine the BMC reusability.

### Solid-Phase Extraction Performance of the Imprinted Polymeric Cartridges

The solid-phase extraction performance of BMC was carried out with BPA solutions prepared using tap water and synthetic wastewater. Tap water (100 mL) and synthetic wastewater solutions (100 mL) containing BPA varying between 0.05 and 0.25 mg/L were prepared and passed through the BMC column. After the adsorption process, 5.0 mL of a 1.0 M NaOH solution was passed through the cartridge for BPA extraction. The column was then washed again with ultrapure water to make it usable again. The synthetic wastewater composition is given in Table S3.

Optimization of experimental conditions and evaluation of extraction efficiency is usually performed by examining enrichment factor (*EF*) and extraction recovery (*ER*).^39^ The first parameter is expressed by dividing the BPA concentration in the extraction solution (*C*_elu_) by the initial concentration (*C*_o_)
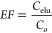
The second parameter is obtained by dividing the adsorbed amount of BPA (*m*_elu_) by the total amount of BPA (*m*_o_) before adsorption. The parameter (*ER*, %) is calculated using the following equation
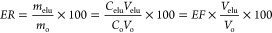
Here, the volumes of extraction solution and adsorption solution are defined by *V*_elu_ and *V*_o_, respectively.

## Results and Discussion

### Characterization

**Figure 2:**
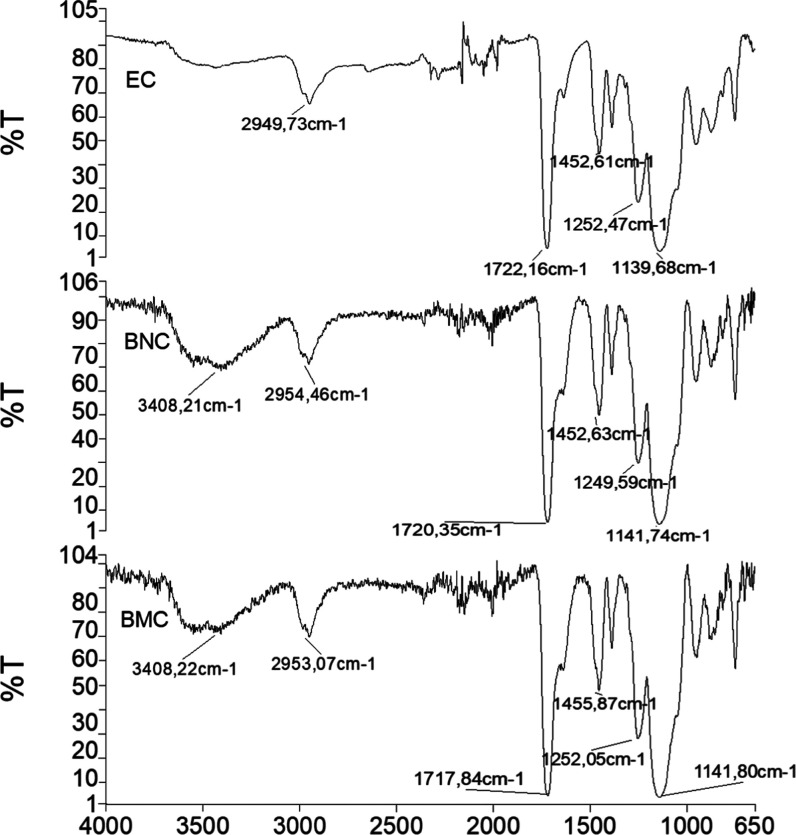
FTIR spectra of the polymeric cartridges.

Elemental analysis and FTIR-ATR analysis were carried out to examine the chemical structures. The possible chemical structure of the BMC and EC is given in Figure S5. FTIR-ATR spectra are given in Figure 2. The structural differences between BMC and BNC from the EC are due to MAPA being used as a functional monomer. When analyzing the spectra, aliphatic C–H bending bands around 2950 cm^–1^, C=O stretching bands around 1700 cm^–1^, and C–O stretching bands around 1145 cm^–1^ are common bands and are due to EDMA used as crosslinkers. For BMC and BNC spectra, C=C stretching bands peaks of 1400 and 1600 cm^–1^ originate from the aromatic ring and aromatic C–H bending bands around 950 cm^–1^ belong to MAPA used as a functional monomer. FTIR-ATR analysis proves that the polymerization was carried out successfully and the functional monomer is incorporated into BMC and BNC structures.

The MAPA amounts in BMC and BNC can be determined according to the elemental analysis results (Table S4). Given the chemical nature of the crosslinker (EDMA) and functional monomer (MAPA) used for the synthesis of polymeric cartridges, the nitrogen element is present only in the MAPA functional monomer. Therefore, nitrogen stoichiometry was applied to the results of elemental analysis. According to the results, it was determined that BMC and BNC were made of 44.7 mmol MAPA/g polymer and 44.2 mmol MAPA/g polymer, respectively. As a result, the presence of MAPA in BNC and BNC was proved by FTIR-ATR analysis, and the MAPA amounts were determined by the elemental analysis results.

The swelling kinetics of the polymeric cartridges were examined by determining the degree of swelling at certain time intervals. The equilibrium swelling results of the polymeric cartridge are given in Figure S6. The results show that the water intake rates of polymeric cartridges are quite high and the percentage of the swelling has reached about 80% within the first 5 min, and it has reached equilibrium after 15 min. In addition, when the swelling ratios and an indication of the rate of water trapping are examined, the low degree of swelling of BMC and BNC according to EC confirms the incorporation of MAPA, a hydrophobic functional monomer, into the structure of BMC and BNC. The equilibrium swelling degree and % porosity values for polymeric cartridges are calculated and given in Table S5. According to the results, the polymeric cartridges are highly porous.

Specific surface area and pore sizes are important properties affecting the adsorption capacity. An automatic surface area and pore size analyzer with nitrogen gas adsorption was used for determining the specific surface areas and pore sizes of the polymeric cartridges. Multipoint BET analysis was applied to the adsorption data for determining the surface area, and BJH analysis was performed to determine the average pore diameter and total pore volume (Table 1). Compared to the literature,^40,41^ it was concluded that the specific surface areas of monolithic columns are high. The pore diameters range from 4 to 14.5 nm and indicate that monolithic columns have a mesopore structure and the pore diameter is suitable for BPA diffusion. With the incorporation of MAPA into the structure, the pore diameters of BMC and BNC decreased and their specific surface areas increased. As the surface area increases, the wettability is expected to increase as the number of active sites on the surface will increase. However, this depends on the functional monomer on the surface. If the hydrophobic functional monomers such as MAPA are used, their wettability is expected to decrease as the surface area increases. These results showed that the specific surface area and pore size analysis is compatible with the swelling kinetics and the polymeric cartridges with high surface area and low back-pressure were prepared. Moreover, their ability to be compressed by 15–20% of their volume without crashing or pulverization reveals their durability and stability due to the high porosity and surface area.

**Table 1:** Surface Area Measurements of Polymeric Cartridges

polymer	average pore diameter^a^ (nm)	total pore volume^b^ (mL/g)	surface area^c^ (m^2^/g)
EC	10.51	2.92	210.08
BNC	9.21	2.52	218.95
BMC	9.56	2.62	222.50

a BJH desorption means the diameter of the pores between 2 and 25 nm.

b BJH total desorption volume of the pores between 2 and 25 nm.

c It was determined by the multipoint BET method.

**Figure 3:**
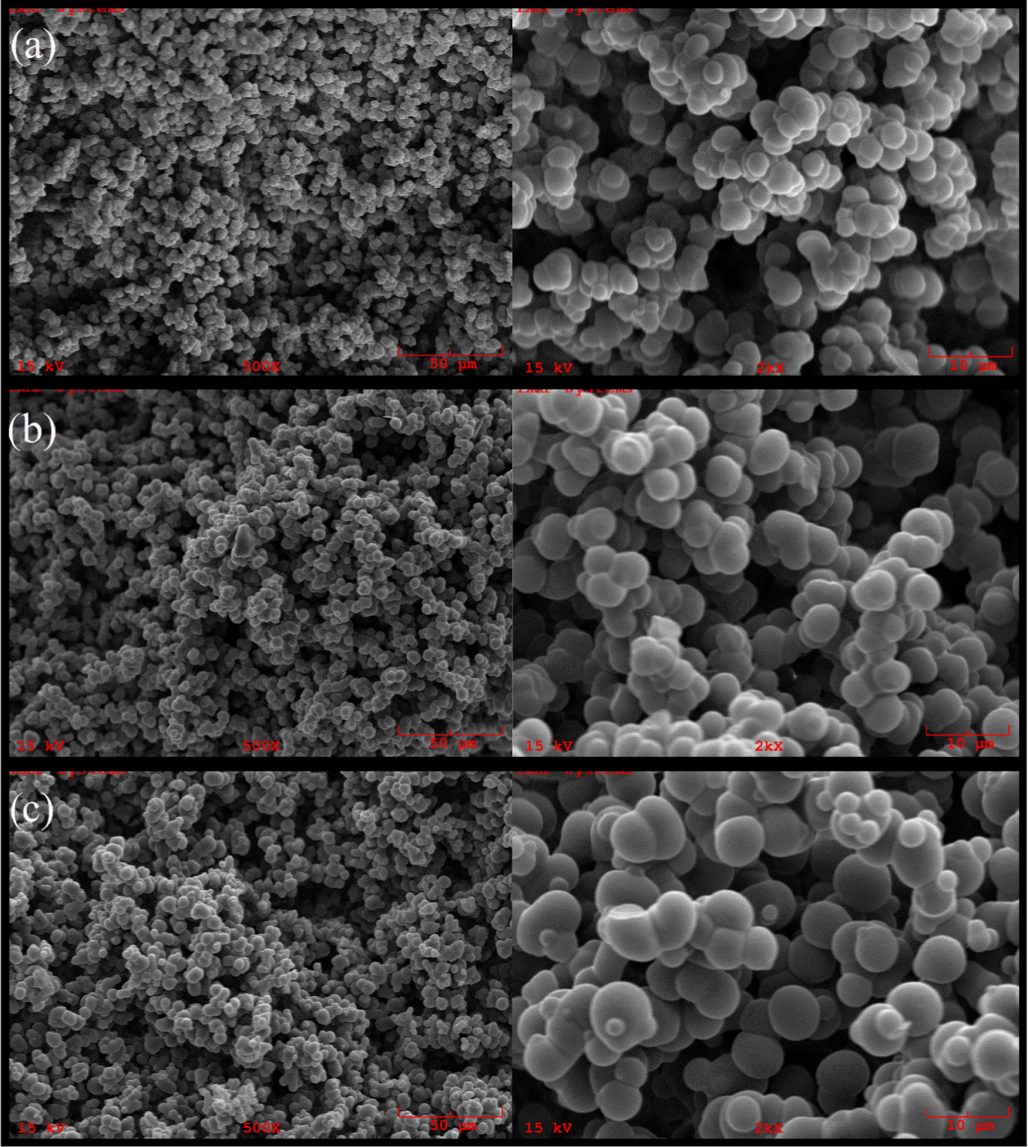
SEM images of BMC (a), BNC (b), and EC (c) at different magnifications (500X and 2kX).

The surface properties and the bulk structures of polymeric cartridges were examined by SEM. SEM images recorded at different magnifications are shown in Figures 3 and S7–S9 (Supporting Information). As seen in Figure 3, the polymeric cartridges have a porous structure and their surfaces are rough.

### Adsorption Studies

**Figure 4:**
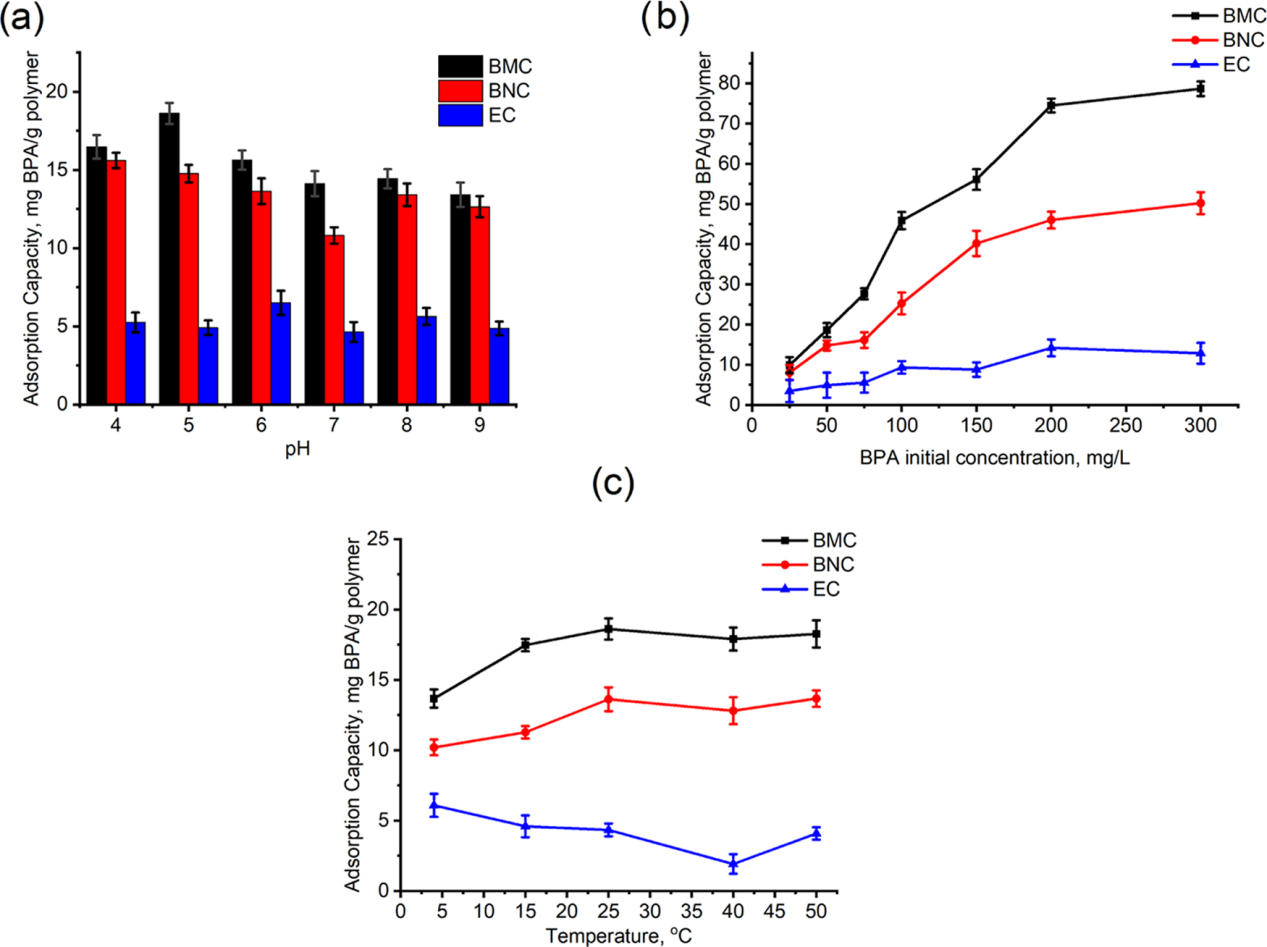
Effect of some parameters on BPA adsorption capacity of polymeric cartridges: (a) pH (*C*_BPA_: 50 mg/L; *V*: 100 mL; temperature: 25 °C; flow rate: 0.75 mL/min; *m*_Polymer_: 0.35 g), (b) BPA initial concentration (*V*: 100 mL; temperature: 25 °C; pH: 5.0; flow rate: 0.75 mL/min; *m*_Polymer_: 0.35 g), and (c) temperature (*C*_BPA_: 50 mg/L; *V*: 100 mL; pH: 5.0; flow rate: 0.75 mL/min; *m*_Polymer_: 0.35 g).

pH is responsible for charged forms of ligand and analyte and the resulting electrostatic interactions. The analyte, BPA, has two p*K*_a_ values for the two ionizable hydroxyl groups at around 9.6 and 11.0. Molecular form and bisphenolate anionic form exist when pH < 9.0 and pH > 9.0, respectively. The pI for amino acid-based functional monomer (MAPA) is around 5.5. The optimum pH was 5.0 according to BPA adsorption capacities varying between pH 4.0–9.0 (Figure 4A). The main interaction was the binding affinity between the analyte and specific binding sites. Considering the uncharged forms of functional monomer and template at the pH, the responsible interactions for the binding in the adsorption process can be considered pi-stacking (π–π interactions) and hydrophobic interaction.

The effect of BPA initial concentration was examined by working with BPA solutions at different concentrations (5–300 mg/L) (Figure 4B). The increasing BPA initial concentration increases the BPA amount diffused to the polymeric cartridge surface and the adsorption capacity of the polymeric cartridges increased depending on BPA initial concentration up to filling all binding sites. The highest capacities were found as 78.7, 50.2, and 14.2 mg/g for BMC, BNC, and EC, respectively. The change in the EC adsorption capacities is only due to the BPA diffusion to the surface and results from nonspecific interactions.

The temperature effect on the BPA adsorption capacity of polymeric cartridges was examined by changing the temperature of the solutions between 4 and 50 °C (Figure 4C). While the adsorption capacities of BMC and BNC polymeric cartridges increased with temperature and reached equilibrium at 25 °C, the adsorption capacity of EC polymeric cartridges decreased with increasing temperature. In the experiments examining the effect of temperature, the highest capacities of BMC and BNC were obtained as 18.5 mg BPA/g polymer and 13.6 mg BPA/g polymer at 25 °C, respectively.

**Figure 5:**
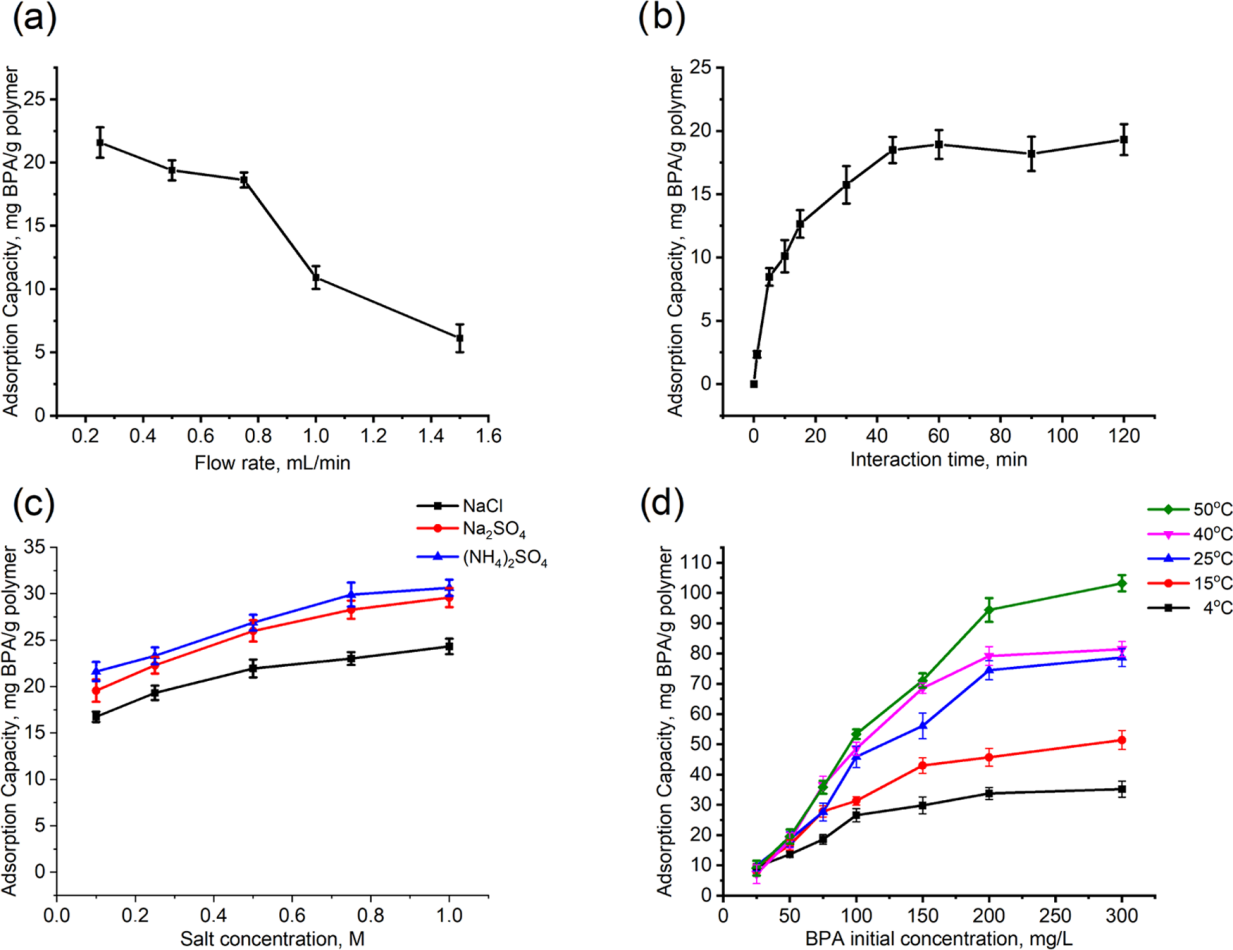
Effect of some parameters on the BPA adsorption capacity of BMC polymeric cartridges: (a) Flow rate (*C*_BPA_: 50 mg/L; *V*: 100 mL; temperature: 25 °C; pH: 5.0; *m*_Polymer_: 0.35 g), (b) interaction time (*C*_BPA_: 50 mg/L; *V*: 100 mL; pH: 5.0; flow rate: 0.75 mL/min; *m*_Polymer_: 0.35 g), (c) salt concentration-type (*C*_BPA_: 50 mg/L; *V*: 100 mL; temperature: 25 °C; pH: 5.0; flow rate: 0.75 mL/min; *m*_Polymer_: 0.40 g), and (d) effect of temperature at different BPA initial concentrations (*V*: 100 mL; pH: 5.0; flow rate: 0.75 mL/min; *m*_Polymer_: 0.40 g).

The flow rate was studied in the range of 0.5–1.5 mL/min to investigate the effect of flow rate on the capacity. The results are given in Figure 5A. At the examined flow rates, the back-pressure of the polymeric cartridge was found to be below 2.0 bar. The optimum flow rate for the adsorption of BPA was determined as 0.75 mL/min considering the adsorption capacity and incubation time. The interaction time of BPA with the surface is reduced by increasing the flow rate, and since the adsorption process does not reach equilibrium, a decrease in the capacity is recorded.

The effect of interaction time on the capacity was examined by determining the capacity at certain times. The change in the capacity of BMC by the interaction time is given in Figure 5B. The adsorption capacity reached equilibrium after 45 min. The BPA adsorption reached equilibrium rapidly because of the high binding affinity between BPA and MAPA and reached a high degree of swelling considering the porous structure of the synthesized polymeric cartridges.

The effect of salts types on BPA adsorption by hydrophobic interactions is explained by the effect of the Hofmeister (or lyotropic) series on the surface tension.^42,43^ In these experiments, the solutions of NaCl, (NH_4_)_2_SO_4_, and Na_2_SO_4_ salts containing BPA were used to examine the changes in BMC capacity (Figure 5C). The adsorbed amount of BPA on the BMC surface increased by increasing the salt concentration. The highest capacity was obtained in the presence of Na_2_SO_4_, and the results were consistent with the Hofmeister series.^44^ These results indicate that the interactions between BPA and MAPA depend on hydrophobicity.

The BMC adsorption capacity and the thermodynamics of BPA adsorption were examined by changing the temperature and the initial BPA concentration together (Figure 5D). In these experiments, the adsorption capacity of 103.2 mg BPA/g was reached with BMC. Increasing the adsorption capacity with temperature indicates that hydrophobic interactions predominate in BPA adsorption.^45,46^ According to these results, it is proved that the BPA adsorption process is specific and occurs by hydrophobic interaction.

**Figure 6:**
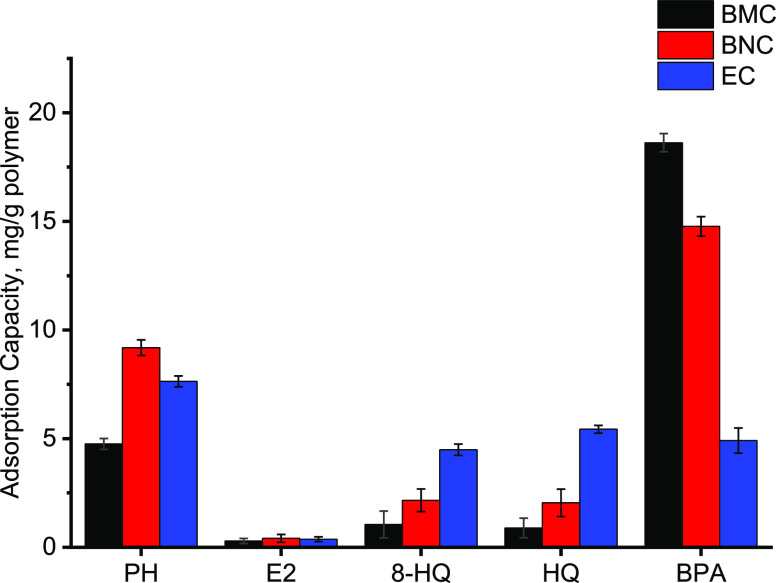
Adsorption capacities of polymeric cartridges for BPA and competitive substances.

To indicate the recognition sites of BPA on the imprinted polymeric cartridge (BMC), the selectivity studies were carried out in an aqueous solution using the competitor molecules which have similar structures to BPA. The solutions containing phenol (PH), hydroxyquinoline (8-HQ), β-estradiol (E2), and hydroquinone (HQ) were prepared and passed through polymeric cartridges under the optimum conditions. The adsorption capacities are given in Figure 6. Table 2 contains distribution coefficients (*k*_D_) and selectivity parameters (*k* and *k*′).

**Table 2:** Selectivity Parameters of Polymeric Cartridges

	polymer				
	BMC		BNC		
molecule	*k*_D_	*k*	*k*_D_	*k*	*k*′
PH	0.14	57.60	0.51	2.55	22.6
E2	0.57	14.63	5.84	0.22	63.9
8-HQ	0.02	362.99	0.05	25.80	14.1
HQ	0.02	434.77	0.05	27.48	15.8
BPA	8.21		1.31		

The selectivity coefficients refer to the ability of the imprinted polymers to recognize the target molecule against competitor molecules. The relative selectivity coefficient refers to how many times the imprinted polymer recognizes the target molecule relative to the nonimprinted polymer. In Table 2, the coefficients indicate that the molecularly imprinted polymeric cartridges include BPA recognition cavities in the molecularly imprinted cartridges (BMC) with size and shape selectivity. Moreover, it was concluded that BMC polymeric cartridges can be successfully used for BPA removal with high selectivity and high capacity from the complex wastewater.

### Reusability

**Figure 7:**
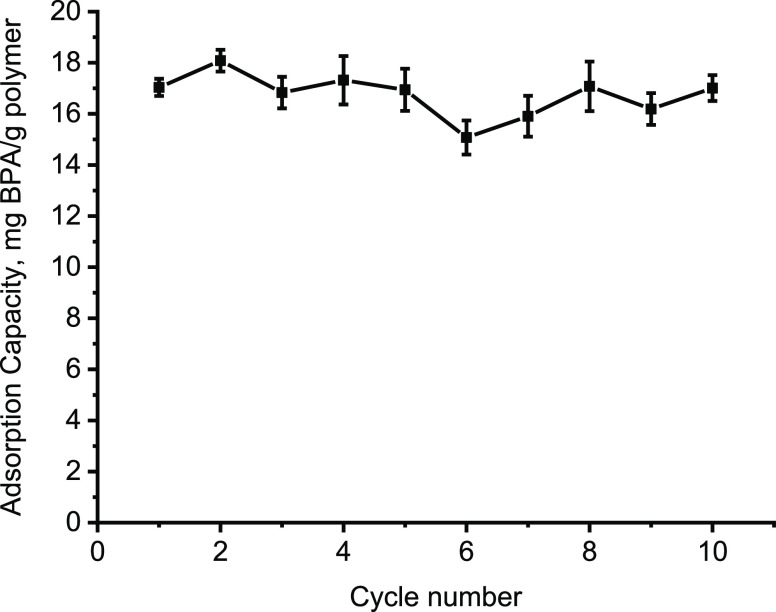
Reusability of BMC polymeric cartridges. *C*_BPA_: 50 mg/L; *V*: 100 mL; flow rate: 0.75 mL/min; pH: 5.0; temperature: 25 °C; *m*_Polymer_: 0.36 g.

Reusability of BMC was performed with 10 adsorption–desorption cycles using the same polymeric cartridges (Figure 7). After 10 cycles, the capacity of the BMC polymeric cartridge decreased by 7%. These results indicate that the cartridges are highly preferable adsorbents for the BPA removal from an aqueous solution by offering cost-effective high reusability.

### Solid-Phase Extraction Performance of the Imprinted Polymeric Cartridge

To investigate the extraction performance of BMC, the enrichment factor (EF) and extraction recovery (ER) values were calculated for BPA extraction in tap water and synthetic wastewater solutions using BMC (Table 3). Extraction recovery (ER) values for BPA extraction in tap water ranged between 92 and 96%, while this value for synthetic wastewater samples ranged from 89 to 94%. Moreover, the calculated enrichment factor (EF) values range from 18.4 to 19.1 for tap water, while this value for synthetic wastewater ranges from 17.9 to 18.8 considering the volume of solutions used in the adsorption–desorption processes. These results indicate that BMC can be successfully applied as an SPE system for the selective removal of BPA from different media.

**Table 3:** BPA Recovery Values from Synthetic Wastewater and Tap Water

polymer	medium	BPA concentration (mg/L)	EF	ER (%)
BMC	synthetic wastewater	0.05	17.9 ± 0.1	89.3 ± 0.5
		0.10	18.8 ± 0.2	93.8 ± 1.0
		0.25	18.3 ± 0.2	91.7 ± 1.0
BMC	tap water	0.05	18.7 ± 0.2	93.8 ± 1.0
		0.10	19.1 ± 0.1	95.7 ± 1.0
		0.25	18.4 ± 0.2	92.2 ± 1.0

To compare the BPA removal performance of the BMC column, the studies for the removal and preconcentration of BPA from various media are summarized in Table S6. Molecular imprinting technology gives selectivity to the adsorbent, and the different functional monomers have been used in the literature to obtain higher selectivity. The most commonly used functional monomers for BPA imprinting are methacrylic acid and 4-vinyl pyridine.^9^ A small number of hydrophobic monomers have been used in the literature for BPA imprinting. In this study, MAPA functional monomer with hydrophobic properties was first used to create recognition sites to increase the efficiency of imprinting. In addition to the parameters such as selectivity, capacity, and SPE usage of the absorber in the removal or preconcentration process, the ease of operation and the cost are also important on an industrial scale in the adsorption applications of analytes such as BPA. Selectivity is important for the determination of the analyte as well as the removal of harmful analytes from complex environments. Therefore, the polymeric cartridge synthesized in addition to its high capacity, its high selectivity, and its successful use in the preconcentration process reveal its superiority over other studies.

### Adsorption Isotherms and Kinetic Model

The rate control step in the adsorption process was examined by applying the pseudo-first-order and pseudo-second-order kinetic models to the adsorption data for the investigations of BPA adsorption kinetics.^47^ The following equation states Lagergren first-order equation also known as the pseudo-first-order rate equation
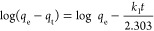
Inequality, *k*_1_ (min^–1^) is the first-order rate constant, and *q*_e_ and *q*_t_ refer to the adsorption capacities at equilibrium and any time, respectively.

The pseudo-second-order adsorption kinetics model expressed by the following equation depends on the equilibrium adsorption capacity
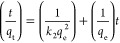
Inequality, *k*_2_ refers to the pseudo-second-order adsorption rate constant (g mg^–1^ min^–1^), and *q*_e_ and *q*_t_ refer to the adsorption capacities at equilibrium and any time, respectively.

The adsorption process is carried out by a multistep mechanism, such as diffusion of the analyte to the surface by mass transfer, diffusion into the pore, and binding of the analyte physically or chemically to the adsorption sites. The pseudo-first-order (diffusion-controlled) and the pseudo-second-order (chemically controlled) kinetic models explain these control mechanisms. The pseudo-first-order kinetic model indicates that the rate-determining step of the adsorption process is the analyte diffusion to the adsorbent surface, while the rate-determining step of the adsorption process is the interaction between the analyte and the adsorbent according to the pseudo-second-order kinetic model. The kinetic parameters for the BPA adsorption process are calculated in Table 4. Consequently, the kinetic parameters indicate that the BPA adsorption on the surface of BMC is chemically controlled without any restriction of diffusion.

**Table 4:** Kinetic Parameters for BPA Adsorption

	experimental	pseudo-first-order kinetics			pseudo-second-order kinetics		
*C*_o_ (mg/L)	*q*_e_ (mg g^–1^)	*k*_1_ (dk^–1^)	*q*_eq_ (mg g^–1^)	*R*^2^	*k*_2_ (mg L^–1^dk^–1^)	*q*_eq_ (mg g^–1^)	*R*^2^
50	19.0	0.084	21.0	0.967	0.068	21.93	0.994

Temkin, Langmuir, Freundlich, and Dubinin–Radushkevich isotherm models were applied to BPA adsorption data examining the adsorption mechanism, surface properties, and adsorption degree.

The Langmuir model assumes that the monolayer adsorption arises from the same or equivalent binding, without any steric hindrance and lateral interactions between the adsorbed molecules. The Langmuir isotherm model equation is shown in Table 5. The following expression defines the separation factor (*R*_L_)^48^
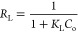
Here, *K*_L_ (L/mg) and *C*_o_ (mg/L) refer to the Langmuir constant and BPA initial concentration, respectively. Low *R*_L_ values indicate that adsorption is more favorable. The *R*_L_ value shows that the adsorption process is unfavorable (*R*_L_ > 1), linear (*R*_L_ = 1), suitable (0 < *R*_L_ < 1), or reversible (*R*_L_ = 0).

**Table 5:** Adsorption Isotherm Models

isotherm	equation	graph
Langmuir		
Temkin		*q*_e_ – ln* C*_e_
Freundlich	log* q*_e_ = log* K*_F_ + 1/*n*log* C*_e_	log *q*_e_*–* log* C*_e_
Dubinin–Radushkevich	ln* q*_e_ = ln* q*_s_ – *k*_ads_ ε^2^	ln* q*_e_ – ε^2^

Freundlich isotherm^49^ is one of the oldest known isotherms, which is not limited to monolayer adsorption and defines reversible and nonideal adsorption. This isotherm model can be utilized to describe the multilayer adsorption where the heat of adsorption on the heterogeneous surface and the affinity distribution are not homogeneous. The linearized equation of the Freundlich isotherm is given in Table 5. In the graph drawn according to the equation, the slope varying between 0 and 1 is the criterion of the adsorption density and surface heterogeneity, and as this value approaches zero, the adsorption becomes more heterogeneous. However, a lower value (<1) means chemisorption, while physical adsorption is defined if the 1/*n* value is above 1.^50^

Temkin isotherm was first used to identify hydrogen adsorption to platinum electrodes in acidic solutions. The isotherm^51^ includes a factor dependent on adsorbent–adsorbate interactions. Omitting excessive low and high concentrations, it assumes that the adsorption temperature in the model layer (the temperature function) will fall linearly instead of logarithmic with the degree of coverage of the surface. The Temkin equation (Table 5) is perfect for estimating gas-phase equilibrium; on the contrary, complex adsorption systems, including the liquid-phase adsorption isotherms, cannot be represented.^52^

The Dubinin–Radushkevich isotherm,^53^ initially, explains an experimental model designed for the gas adsorption to the microporous solids following the pore-filling mechanism. According to this model, the adsorption mechanism is applied to a heterogeneous surface with the Gaussian energy distribution.^54^ The model frequently adapts to high adsorption activity and adsorption data at medium concentration ranges. The model was generally used to distinguish the chemical and physical adsorption of metal ions, using the average free energy and energy per analyte molecule (energy required for the desorption in the area of adsorption). The Dubinin–Radushkevich isotherm model equation is given in Table 5. The ε parameter in the Dubinin–Radushkevich equation is defined as
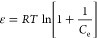
Inequality, *R* refers to the gas constant (8.314 J/mol·K), *T* is the adsorption temperature (K), and *C*_e_ represents the analyte concentration (mg/L) at equilibrium.

The graphs obtained by applying the adsorption isotherm models are given in Figures S10–S13. The slopes and intercepts of lines were used to calculate the isotherm constants, which are given in Table 6. According to the regression coefficients (*R*^2^) of the adsorption models, the adsorption of BPA on BMC surfaces corresponds to the Langmuir adsorption model. Moreover, the maximum capacity values obtained from the Langmuir isotherm are also consistent with the experimental data obtained for BPA adsorption. The Langmuir isotherm explains that the binding sites that perform the adsorption of BPA on BMC have a homogeneous, same energy level and binding affinity and that BPA adsorption is limited to a single layer. The *R*_L_ values obtained from the Langmuir isotherm also vary from 0 to 1, showing that the adsorption process is convenient.

**Table 6:** Calculated Isotherm Constants for BPA Adsorption

	temperature									
isotherm model	4 °C		15 °C		25 °C		40 °C		50 °C	
experimental	*Q* (mg/g)	35.2	*Q* (mg/g)	51.4	*Q* (mg/g)	78.7	*Q* (mg/g)	87.2	*Q* (mg/g)	101.2
Langmuir	*K*_L_ (L/mg)	0.043	*K*_L_ (L/mg)	0.078	*K*_L_ (L/mg)	0.094	*K*_L_ (L/mg)	0.107	*K*_L_ (L/mg)	0.077
	*Q*_o_ (mg/g)	38.0	*Q*_o_ (mg/g)	52.9	*Q*_o_ (mg/g)	88.5	*Q*_o_ (mg/g)	98.1	*Q*_o_ (mg/g)	125.0
	*R*_L_	0.48	*R*_L_	0.34	*R*_L_	0.30	*R*_L_	0.27	*R*_L_	0.34
	*R*^2^	0.990	*R*^2^	0.990	*R*^2^	0.994	*R*^2^	0.991	*R*^2^	0.995
Freundlich	1/*n*	0.32	1/*n*	0.32	1/*n*	0.50	1/*n*	0.52	1/*n*	0.59
	*K*_F_	6.5	*K*_F_	10.4	*K*_F_	11.2	*K*_F_	12.1	*K*_F_	11.7
	*R*^2^	0.934	*R*^2^	0.952	*R*^2^	0.927	*R*^2^	0.851	*R*^2^	0.826
Dubinin–Radushkevich	*q*_s_ (mg/g)	28.5	*q*_s_ (mg/g)	34.9	*q*_s_ (mg/g)	49.1	*q*_s_ (mg/g)	62.6	*q*_s_ (mg/g)	68.9
	*K*_ad_ (mol^2^/kJ^2^)	7 × 10^–6^	*K*_ad_ (mol^2^/kJ^2^)	5 × 10^–7^	*K*_ad_ (mol^2^/kJ^2^)	7 × 10^–7^	*K*_ad_ (mol^2^/kJ^2^)	1 × 10^–6^	*K*_ad_ (mol^2^/kJ^2^)	9 × 10^–7^
	*E* (kJ/mol)	1.9 × 10^3^	*E* (kJ/mol)	1 × 10^3^	*E* (kJ/mol)	8.5 × 10^2^	*E* (kJ/mol)	7.1 × 10^2^	*E* (kJ/mol)	7.5 × 10^2^
	*R*^2^	0.765	*R*^2^	0.666	*R*^2^	0.696	*R*^2^	0.8263	*R*^2^	0.800
Temkin	*b*_T_	3.6 × 10^2^	*b*_T_	3.0 × 10^2^	*b*_T_	1.5 × 10^2^	*b*_T_	1.3 × 10^2^	*b*_T_	1.1 × 10^2^
	*A*_T_ (L/g)	0.91	*A*_T_ (L/g)	2.32	*A*_T_ (L/g)	1.23	*A*_T_ (L/g)	1.13	*A*_T_ (L/g)	0.93
	*B* (J/mol)	6.48	*B* (J/mol)	8.15	*B* (J/mol)	17.36	*B* (J/mol)	21.05	*B* (J/mol)	25.77
	*R*^2^	0.956	*R*^2^	0.960	*R*^2^	0.953	*R*^2^	0.935	*R*^2^	0.976

The thermodynamic parameters (Δ*G*^o^, Δ*H*^o^, and Δ*S*^o^) are needed to investigate the thermodynamics of adsorption. For the adsorption process, the thermodynamic parameters are extracted from equilibrium constants (thermodynamic equilibrium constant, *K*_o_) that change with temperature,^55−57^ and *K*_o_ is defined as follows
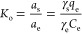
Here, *a*_s_ is the activity of the adsorbed analyte, *a*_e_ is the analyte activity in the equilibrium solution, *q*_e_ is the surface concentration of the BPA (mmol/g polymer), *C*_e_ (mmol/mL) refers to the BPA concentration at the adsorption equilibrium, γ_s_ represents the adsorbed analyte activity coefficient, and γ_e_ denotes the activity coefficient of the analyte at the adsorption equilibrium. As the BPA concentration is close to zero, the activity coefficient is close to 1 and if the equation is adjusted again
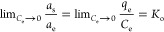
*C*_e_ is plotted against ln(*q*_e_/*C*_e_), and *K*_o_ values are obtained from *C*_e_ zero extrapolation. For different temperatures, *K*_o_ values obtained from the graphs drawn are given in Table S7.

Gibbs free energy change (Δ*G*°) is an indication of whether the change occurs spontaneously and is calculated for the adsorption process as follows
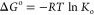
Inequality, *R* and *T* denote the universal gas constant (8,314 J/mol·K) and the adsorption temperature in Kelvin, respectively. Δ*H*^o^ (enthalpy change) provides information about energy release (exothermic process), or consumption (endothermic process) during the adsorption process. Another thermodynamic parameter, Δ*S*^o^ (entropy change), indicates the irregularity during the adsorption process. These parameters are calculated by the integrated van’t Hoff equation.
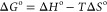

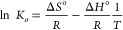
where Δ*H*^o^ is the standard enthalpy change (kJ/mol) and Δ*S*^o^ refers to the standard entropy change (J/mol·K). Δ*H*^o^ and Δ*S*^o^ values are obtained from the slope and intercept by plotting ln*K*_o_ against 1/*T*, respectively.

The adsorption of BPA by BPA-imprinted polymeric cartridges was thermodynamically investigated, and the parameters are given in Table 7. As seen from the table, Δ*G*° values are negative at all temperatures, indicating that BPA adsorption occurs spontaneously, and BPA adsorption is more favorable (increased affinity for BPA) at high temperatures as evidenced by more negative Δ*G*° values. The positive Δ*H*° value defines that the BPA adsorption process is endothermic, and this also explains the increased BPA adsorption with temperature increase. Δ*S*° value indicates the increase in irregularity during the BPA adsorption due to the transformation of the regular water molecules around the hydrophobic groups into an irregular structure after the adsorption process. The Δ*H*° magnitude may also indicate the adsorption type. While the energy generated during physisorption is 2.1–20.9 kJ/mol, the heat of chemisorption is generally over the range of 80–200 kJ/mol. In general, Δ*G*° is over the ranges of −20 to 0 and −80 to −400 kJ/mol for physisorption and chemisorption, respectively.^58^ Therefore, the resulting thermodynamic parameters also support the specificity of BPA adsorption by hydrophobic interactions (physisorption).

**Table 7:** Thermodynamic Quantities Calculated for BPA Adsorption

temperature (°C)		Δ*G*° (kJ/mol)	
4		–1.21	
15		–2.40	
25		–3.48	
40		–5.11	
50		–6.19	
Δ*H*° (kJ/mol)	28.80	Δ*S*° (J/mol·K)	108.27

## Conclusions

In this study, novel molecularly imprinted polymeric cartridges (BMC) were prepared for the selective removal and preconcentration of BPA. Structural characterizations were performed through FTIR-ATR and elemental analysis. In BMC and BNC, the inclusion of functional monomer (MAPA) has been proven by FTIR and the amount of MAPA was determined by elemental analysis as 44.7 mmol/g polymer and 44.2 mmol/g polymer in BMC and BNC, respectively. The surface morphology and properties were examined by the swelling test, SEM, and BET surface area measurements. After characterization, the highest adsorption capacity of BMC was 103.2 mg BPA/g polymer under optimum conditions (pH, flow rate, temperature, etc.). The selectivity and relative selectivity coefficient values in the selectivity studies using the structural analogues indicate that BMC with BPA recognition regions were successfully synthesized not only with high selectivity but also with high capacity. The SPE performance of BMC was carried out using tap water and synthetic wastewater containing BPA and the recovery of extractions ranged from 92 to 96 and 89 to 94% in tap water and synthetic wastewater samples, respectively. Moreover, EF values were calculated as 18.4–19.1 for tap water and 17.9–18.8 for synthetic wastewater. The resulting thermodynamic parameters also support the specificity of BPA adsorption by hydrophobic interactions (physical adsorption). BPA adsorption was consistent with the pseudo-second-order kinetic pattern, which predicted chemical control, without any diffusion restriction on the polymeric cartridge surface. The selectivity is important for the determination of the analyte as well as the removal of harmful analytes from complex environments. In addition to the parameters such as selectivity, capacity, and SPE usage of the absorbent in the removal or preconcentration process, the ease of operation and cost are also important on an industrial scale in the adsorption applications of analytes such as BPA. Therefore, the polymeric cartridge synthesized besides its high capacity, its high selectivity, and its successful use in the preconcentration process reveal its superiority over other similar studies.

## Supporting Information Available

Template–functional monomer (BPA-MAPA) ratio determination; contents of monomers for the polymeric cartridges fabrication; synthetic wastewater composition; equilibrium swelling results, and SEM images recorded at different magnifications of the polymeric cartridges and the graphs obtained by applying the adsorption isotherm models (PDF)
