# Supplementary material for: Investigation of Thermodynamic, Kinetic, and Isothermal Parameters for the Selective Adsorption of Bisphenol A
Source: ACS Omega. 2022 May 24;7(22):18940–52. doi: 10.1021/acsomega.2c01975 (PMC9178953; doi:10.1021/acsomega.2c01975)
Supplement: Supplementary file 1 — ao2c01975_si_001.pdf [file ao2c01975_si_001.pdf]

SUPPORTING INFORMATION

The investigation of thermodynamic, kinetic, and isothermal parameters for the selective adsorption of Bisphenol A

Recep Üzek[a], Serap Şenel[a], Adil Denizli[a]*

[a] Hacettepe University, Faculty of Science, Department of Chemistry, 06800, Ankara, Turkey

Experimental

MAPA, having the structural similarity and the ability to interact strongly with BPA was chosen as a functional monomer to prepare molecularly imprinted polymeric cartridges. The functional monomer/target molecule ratio should be determined in the imprinting selectivity and efficiency. Intermolecular interactions cause changes in the absorbance profiles of molecules in the absorption spectra. Therefore, the differences occur between the absorption spectra of the pure solutions and the absorption spectra of the pre-complexes. In the analysis of BPA-MAPA interaction, MAPA and BPA solutions were prepared by using 3% ethanol-water solution by volume in a total volume of 4 mL with different proportions, and the absorption spectra were recorded.

Table S1. The amount of MAPA and BPA in the pre-complex solution.

**Solution**	**BPA (μmol)**	**MAPA (μmol)**
BPA1	1	-
BPA1-MAPA1	1	1
BPA1-MAPA2	1	2
BPA1-MAPA3	1	3
BPA1-MAPA4	1	4
MAPA1	-	1
MAPA2	-	2
MAPA3	-	3
MAPA4	-	4

UV-visible absorption spectra were recorded in the 200-400 nm range. In order to determine the most suitable preliminary complex ratio in the imprinting, the difference spectra were formed by the difference between the spectra of pure and the pre-complex solutions. The difference spectra are given in Figure S1. After MAPA and BPA interaction, changes in

absorption bands at 280 nm were observed. These changes were taken into consideration in the synthesis of imprinted polymeric cartridges with a 1:4 ratio (BPA1-MAPA4).

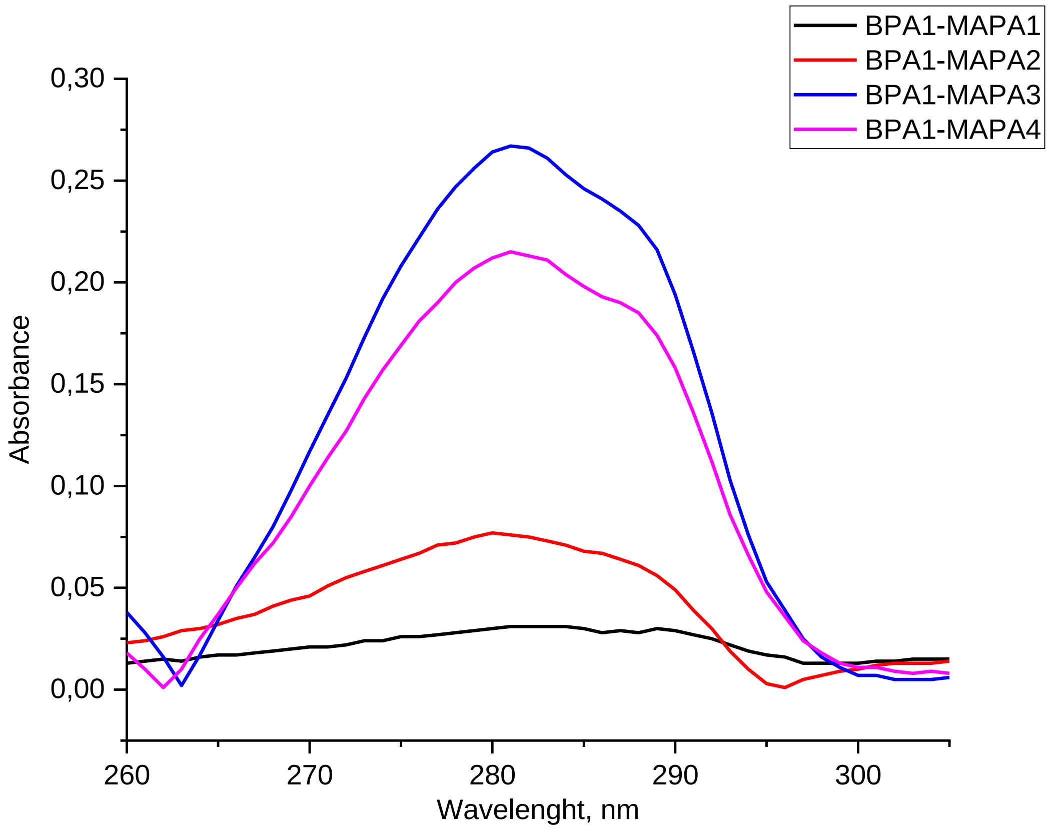

Figure S1. The absorbance spectra created by the interaction of MAPA and BPA with different ratios.

Table S2. The amount of chemicals used in the synthesis of polymeric cartridges.

Polymer	EtOH, mL	EDMA, mL	MAPA, mL	BPA, mg	AIBN, mg
**BMC**	2,600	0,900	1,075	90	22,5
**BNC**	2,600	0,900	1,075	-	22,5
**EC**	2,600	0,900	-	-	22,5

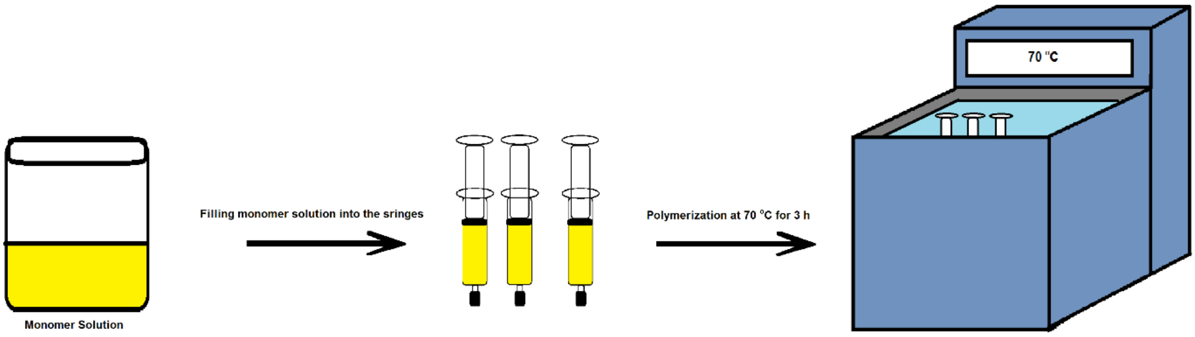

Figure S2. Preparation of polymeric cartridges.

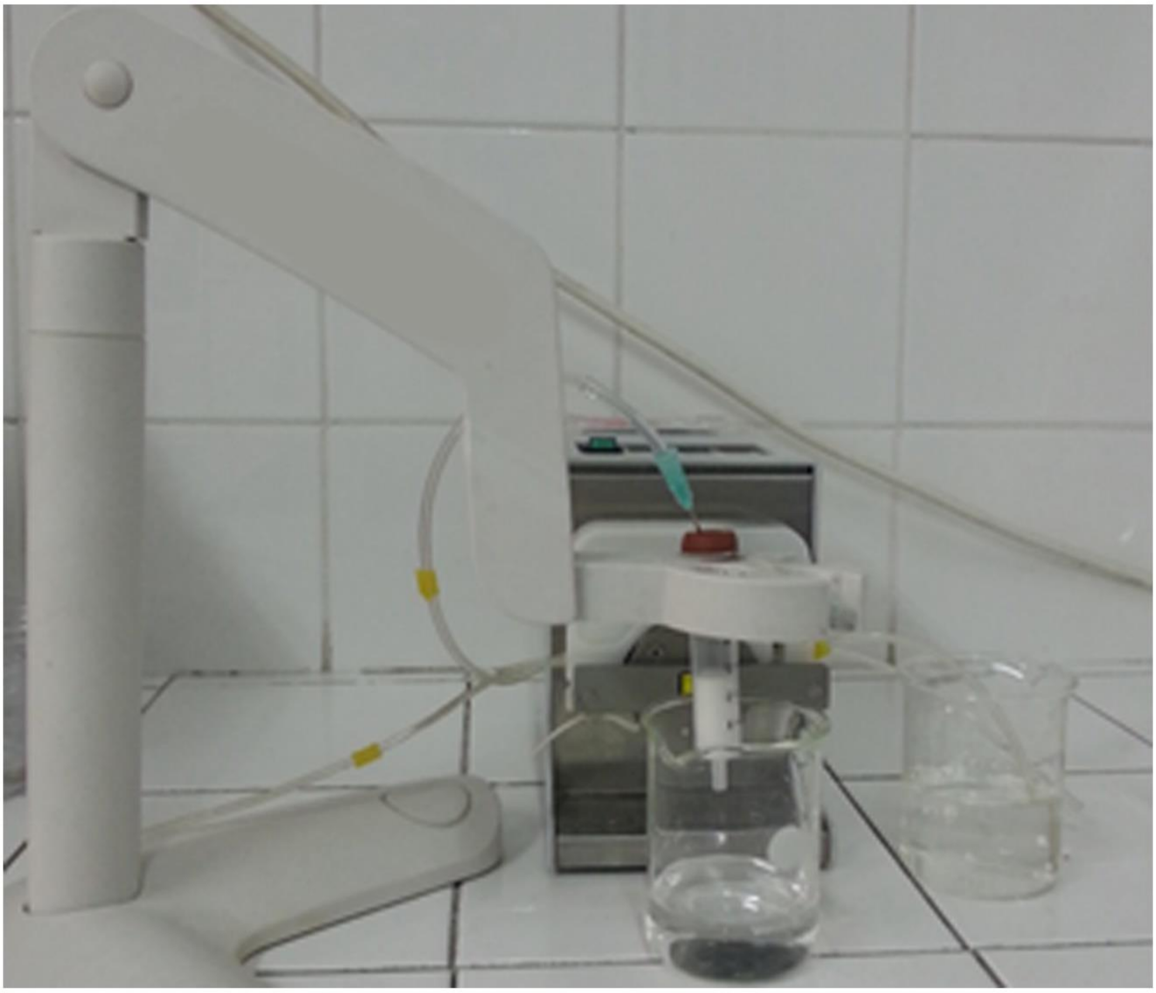

Figure S3. The experimental setup for BPA adsorption.

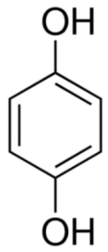

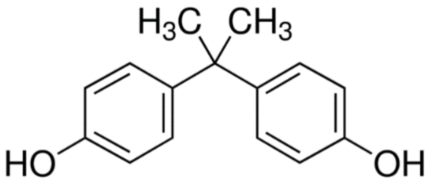

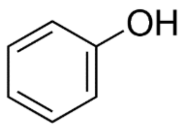

Hydroquinone (110 g/mol)

Bisphenol A (228 g/mol)

Phenol (94 g/mol)

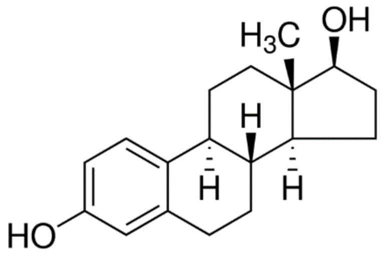

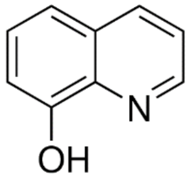

β-Estradiol (272 g/mol)

8-Hydroxyquinone (145 g/mol)

Figure S4. The chemical structures and molar masses of BPA and competing substances.

Table S3. The composition of synthetic wastewater.

**Compound**	**Concentration, mg/L**
NaCl	7.00
$CaCl_2$	10.00
$MnSO_4$	0.038

$MgSO_4$	25.00
$ZnSO_4$	0.035
$NaHCO_3$	125.00
KH_2PO_4	43.94
$FeCl_2$	0.375

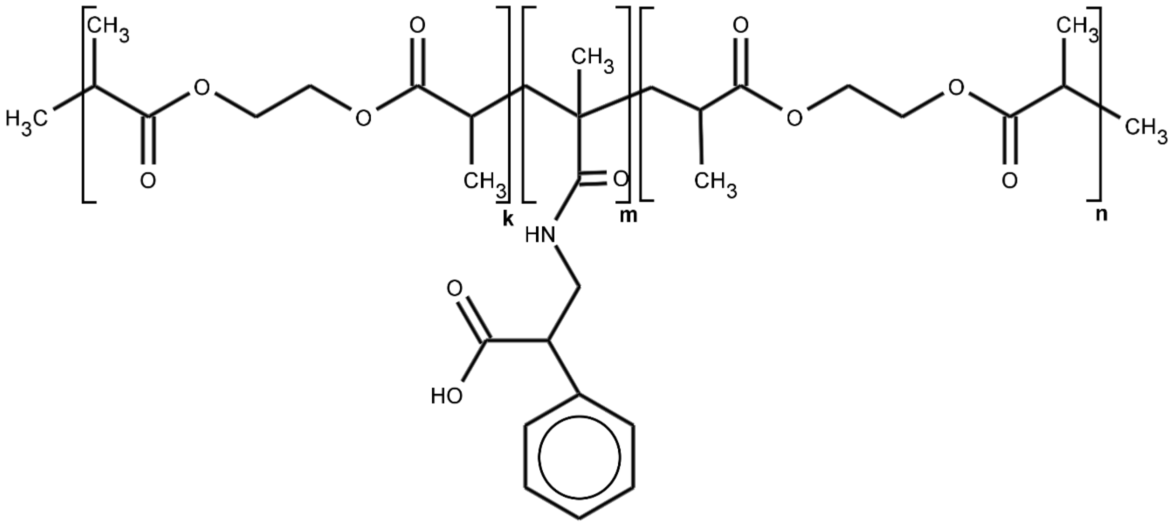

BMC

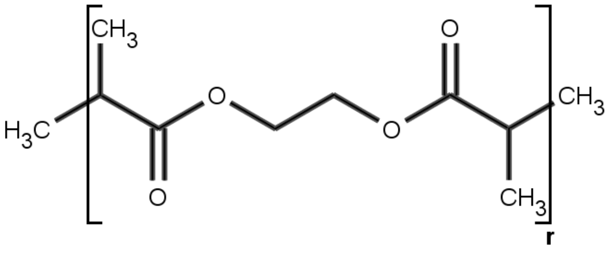

EC

Figure S5. The possible chemical structure of BMC and EC polymeric cartridges.

Table S4. Elemental analysis results of polymers (by mass %).

Polymer	N, %	C, %	H, %	S, %
EC	-	59.04	7.20	-
BNC	0.62	59.86	7.12	-
BMC	0.62	58.17	6.95	-

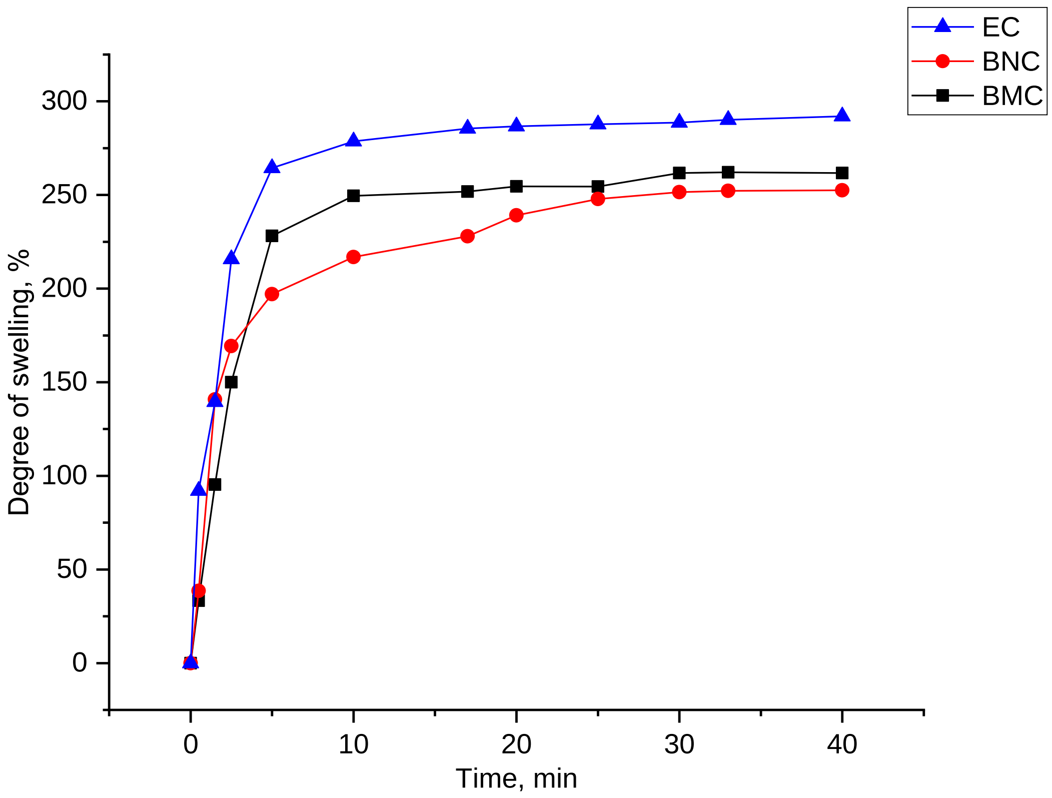

Figure S6. The swelling degree of polymeric cartridges.

Table S5. The equilibrium swelling degree and % porosity values of polymeric cartridges.

Polymer	Swelling degree, %	Porosity, %
EC	292.0	66.4
BNC	252.5	60.7
BMC	261.7	61.9

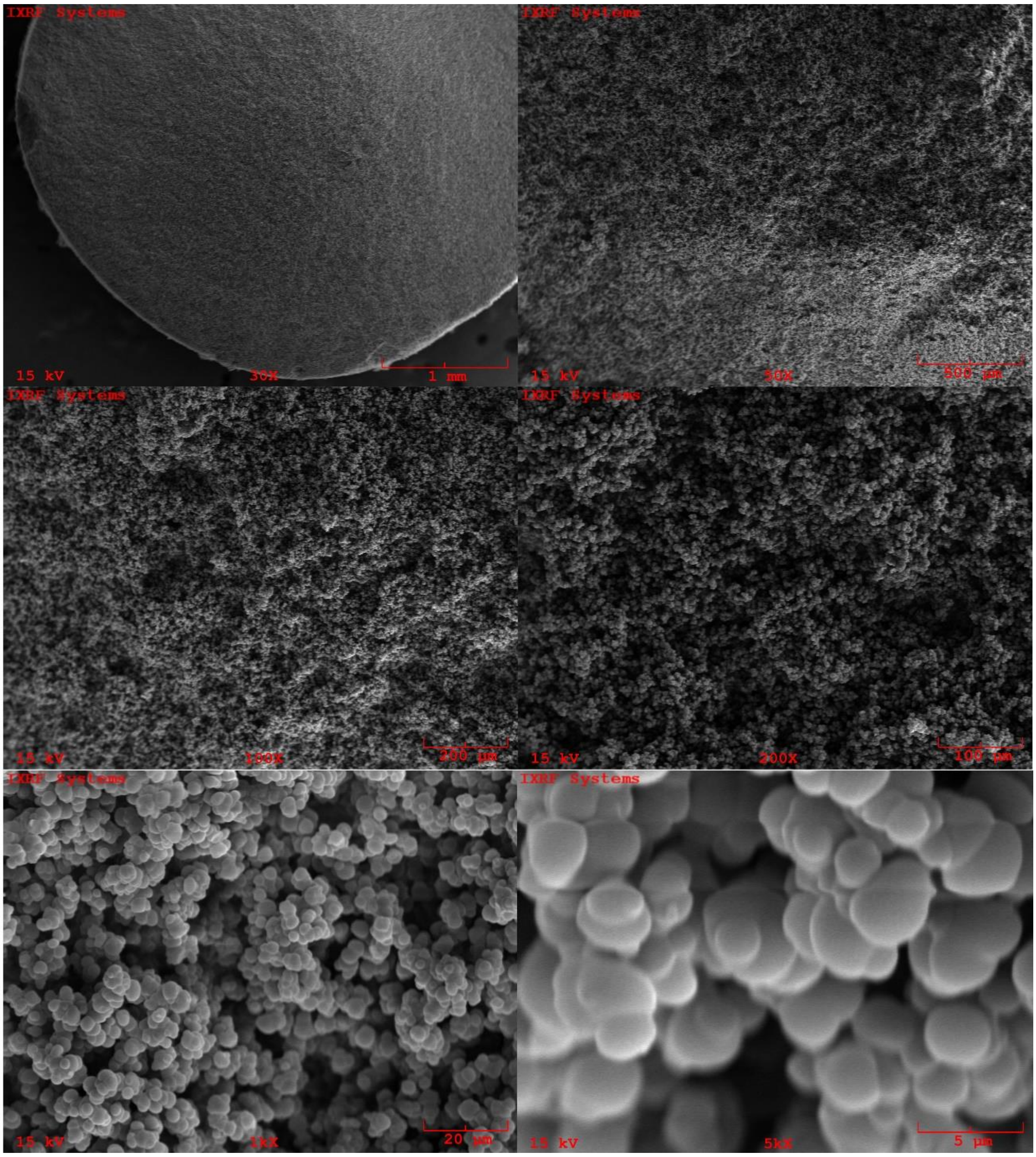

Figure S7. The SEM images at different magnifications of BMC.

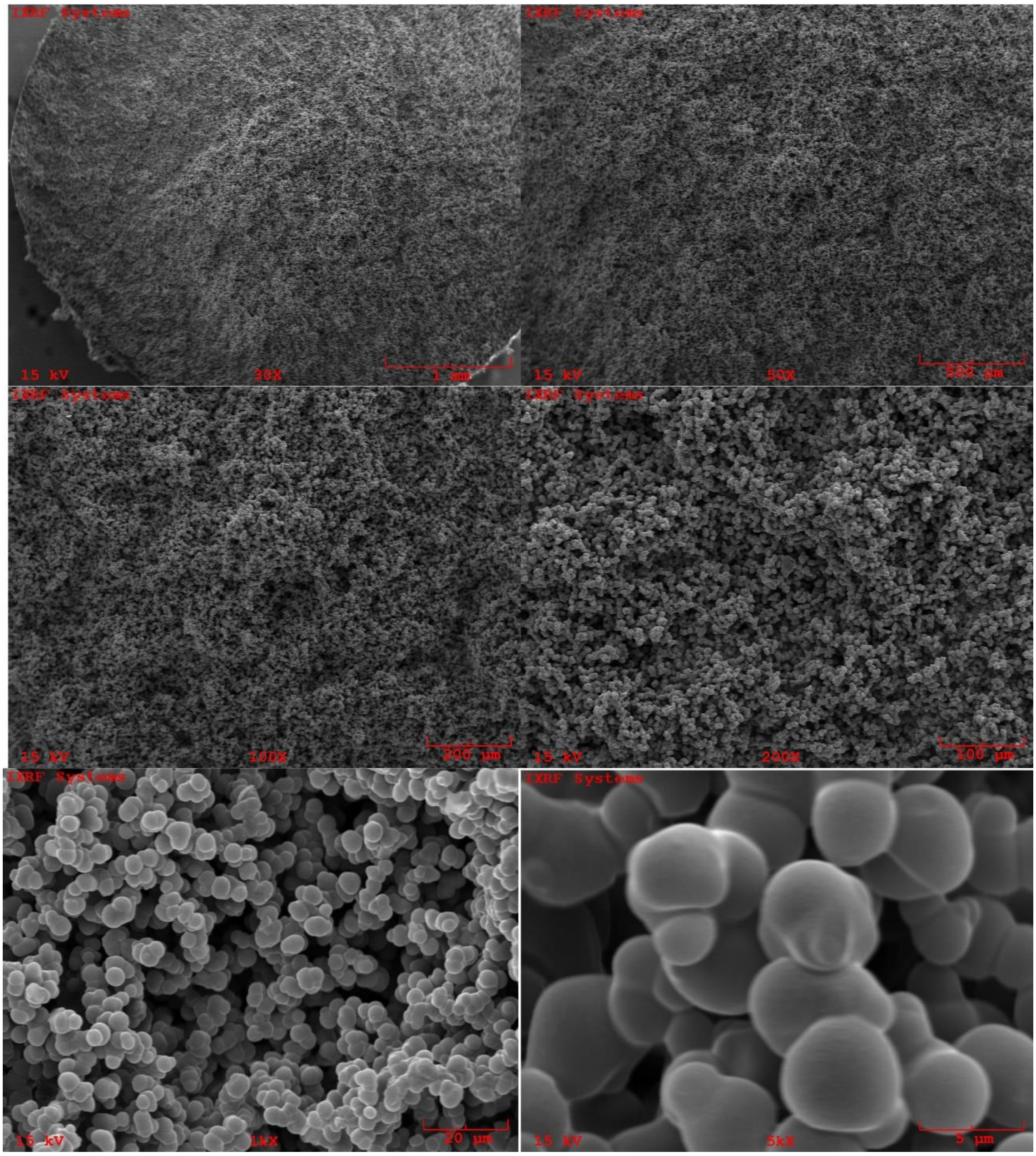

Figure S8. The SEM images at different magnifications of BNC.

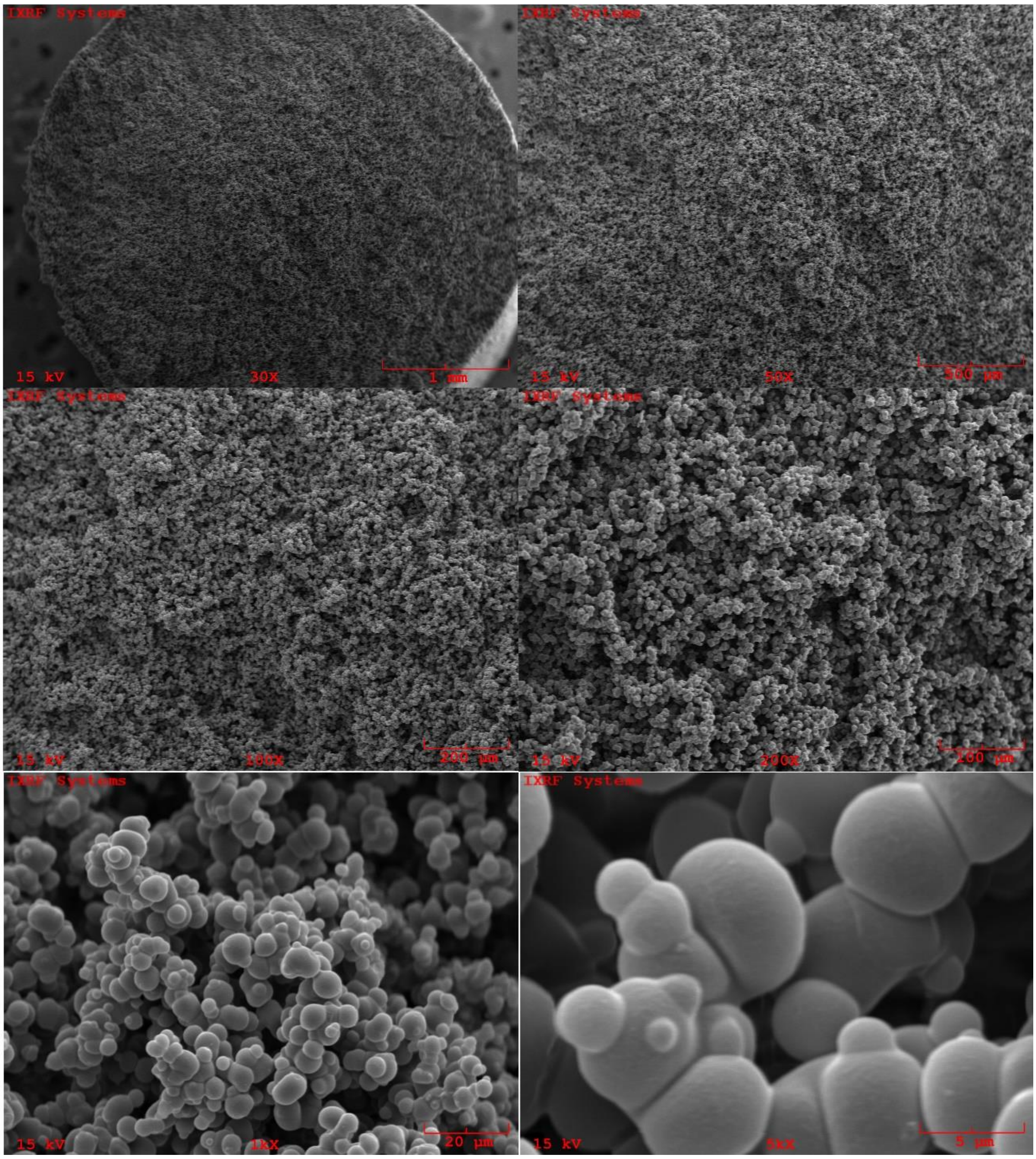

Figure S9. The SEM images at different magnifications of EC.

Table S6. The studies on the removal and pre-concentration of BPA based on adsorption from various media in the literature.

Adsorbent	Method	Functional monomer*	Selectivity	SPE	Real sample	Adsorption capacity, mg/g	[R]
Palygorskite	MIP	4-VP	+	-	-	41.55	1
Zeolite	Adsorption	CTMAB	-	-	-	37.85	2
Granular activated carbon	Adsorption	-	-	-	-	3.54	3
Zeolite	Adsorption	HDTMAB	-	-	-	6.90	4
Polyethersulfone	Adsorption	SPESPE	-	-	-	45.60	5
Porous metal-organic frameworks MIL-101(Cr) and MIL-100(Fe)	Adsorption	-	-	-	-	252.5-55.6	6
Calcium alginate microparticles	Adsorption	MWCNTs	-	-	-	24.53	7
GO	MIP	AMPS-St	+	-	+	84.06	8
Mesoporous silica	Adsorption	CTMAB	-	-	-	416.7	9
m-poly(EDMA-MATrp) microparticles	Adsorption	MATrp	-	-	-	82.4	10
Magnetic montmorillonite	Adsorption	HDTMAB	-	+	+	59.17	11
Chitosan (synthesized) and Chitosan (commercial)	Adsorption	-	-	-	-	34.48-27.02	12
Magnetic MIP	MIP	MAA-CD	+	+	+	11.23	13
Mesoporous silica	MIP	MPABA	+	-	+	6.17	14
Kaolin/Fe_3O_4 composites	MIP	MAA	+	-	+	142.9	15
Organic-inorganic clay	Adsorption	ODTMA	-	-	-	109.89	16
Temperature sensitive MIP	MIP	4-VP-NIPAM	+	+	+	7.86	17

Reduced-GO	Adsorption	-	-	-	-	94.06	[18]
Polyaniline coated Fe_3O_4 nanoparticles	Adsorption	Polyaniline	-	-	-	23.09	[19]
Geotite-activated carbon composite	Adsorption	-	-	-	-	13.60	[20]
MIP polymeric cartridge (BMC)	MIP	MAPA	+	+	+	103.20	This study

*4-VP: 4-vinylpyridine; CTMAB: cetyltrimethyl ammonium bromide; HDTMAB: hexadecyltrimethyl ammonium bromide; SPESPE: Sulfonated polyethylsulfonphenylethane; MWCNTs: Multi-walled carbon nanotubes; AMPS: 2-acrylamido-2-methylpropanesulfonic acid; St: Styrene; GO: Graphene oxide; MATrp: N-methacryloyl-L-tryptophanmethylester; MAA: Methacrylic acid; ODTMA: octadecyltrimethylammonium bromide; CD: cyclodextrin; MPABA: 4-(4-methacryloyloxy-phenylazo)-benzoic acid; MAPA: N-methacryloyl-L-phenylalanine.

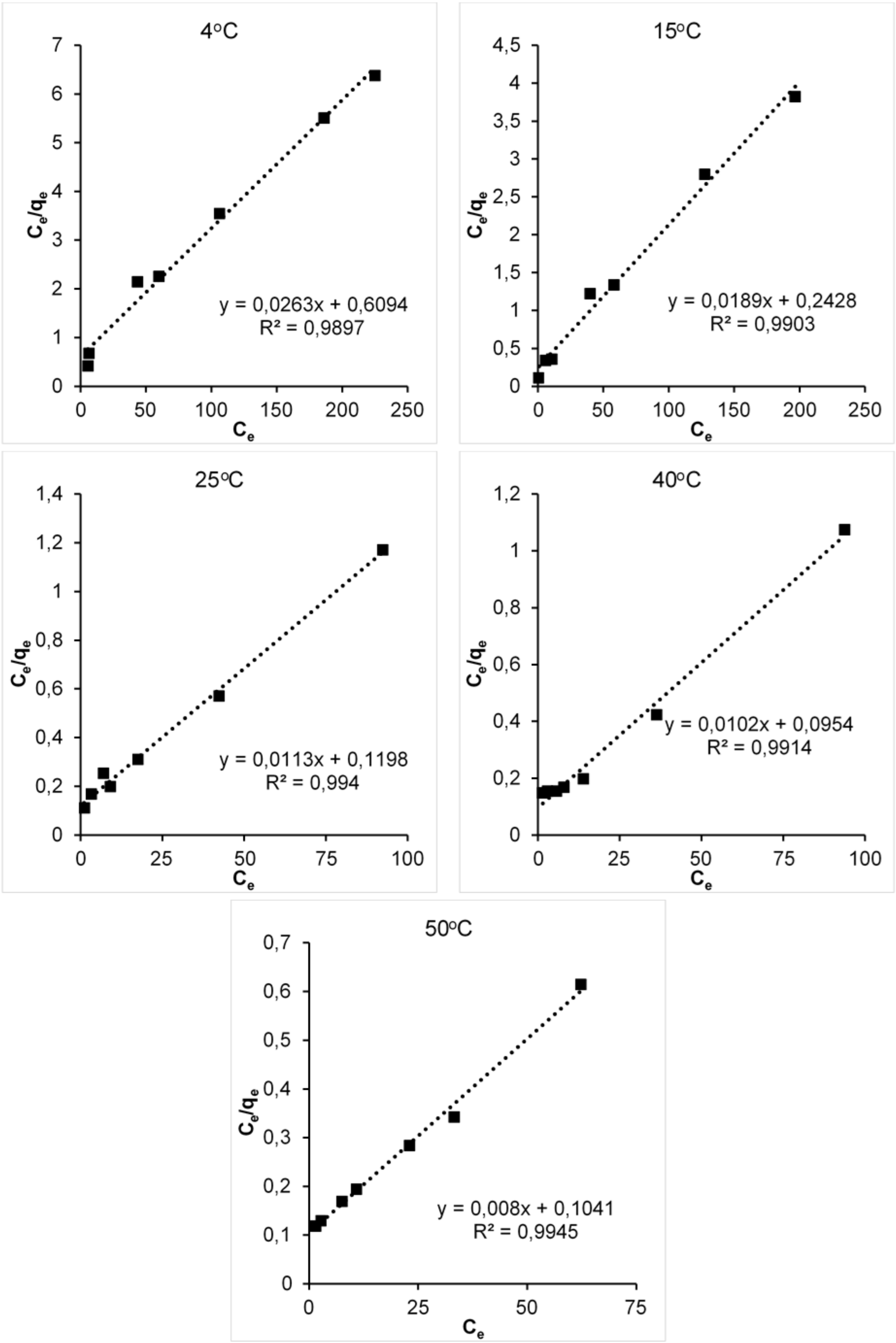

Figure S10. Application of Langmuir isotherm model to adsorption data obtained at different temperatures.

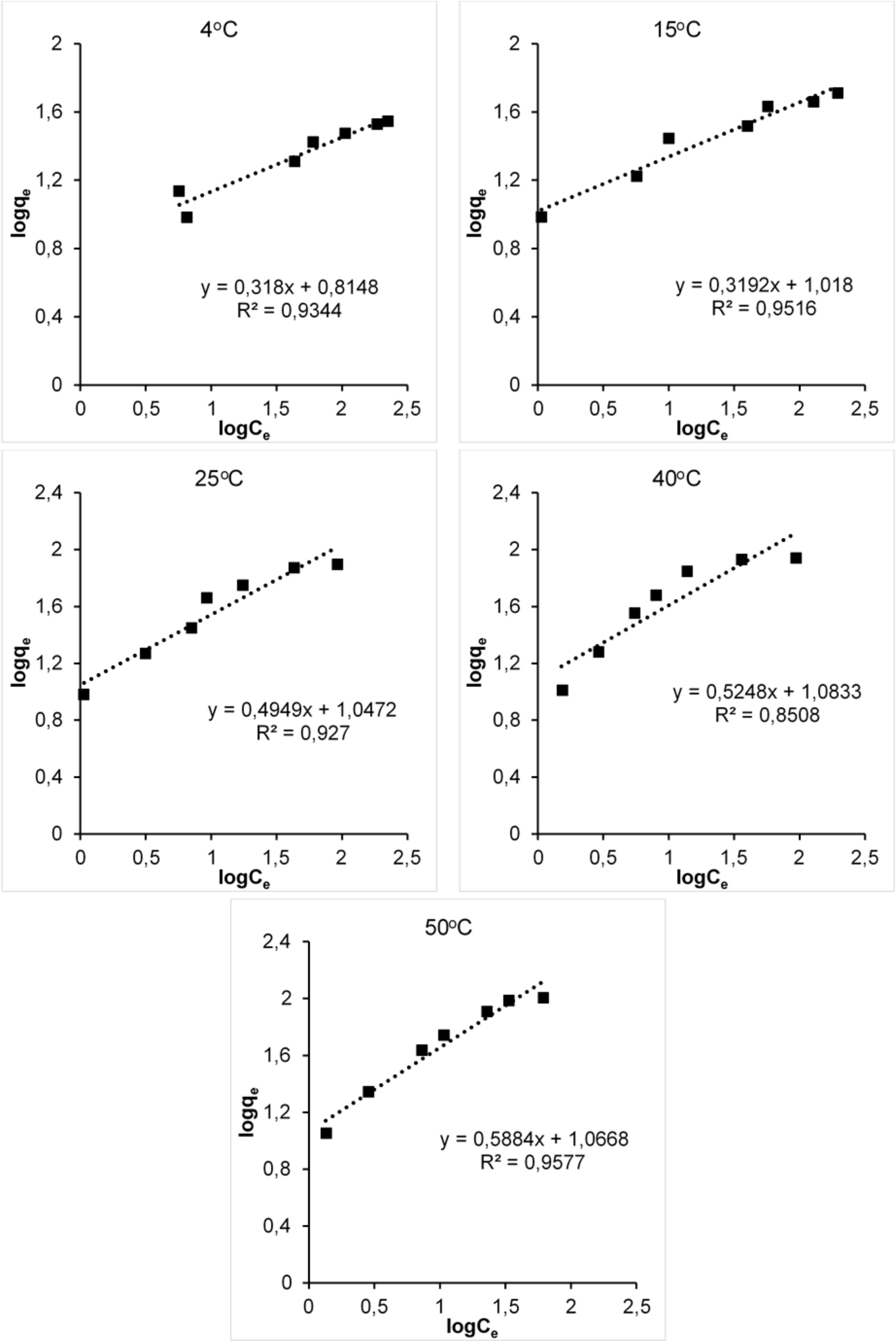

Figure S11. Application of Freundlich isotherm model to adsorption data obtained at different temperatures.

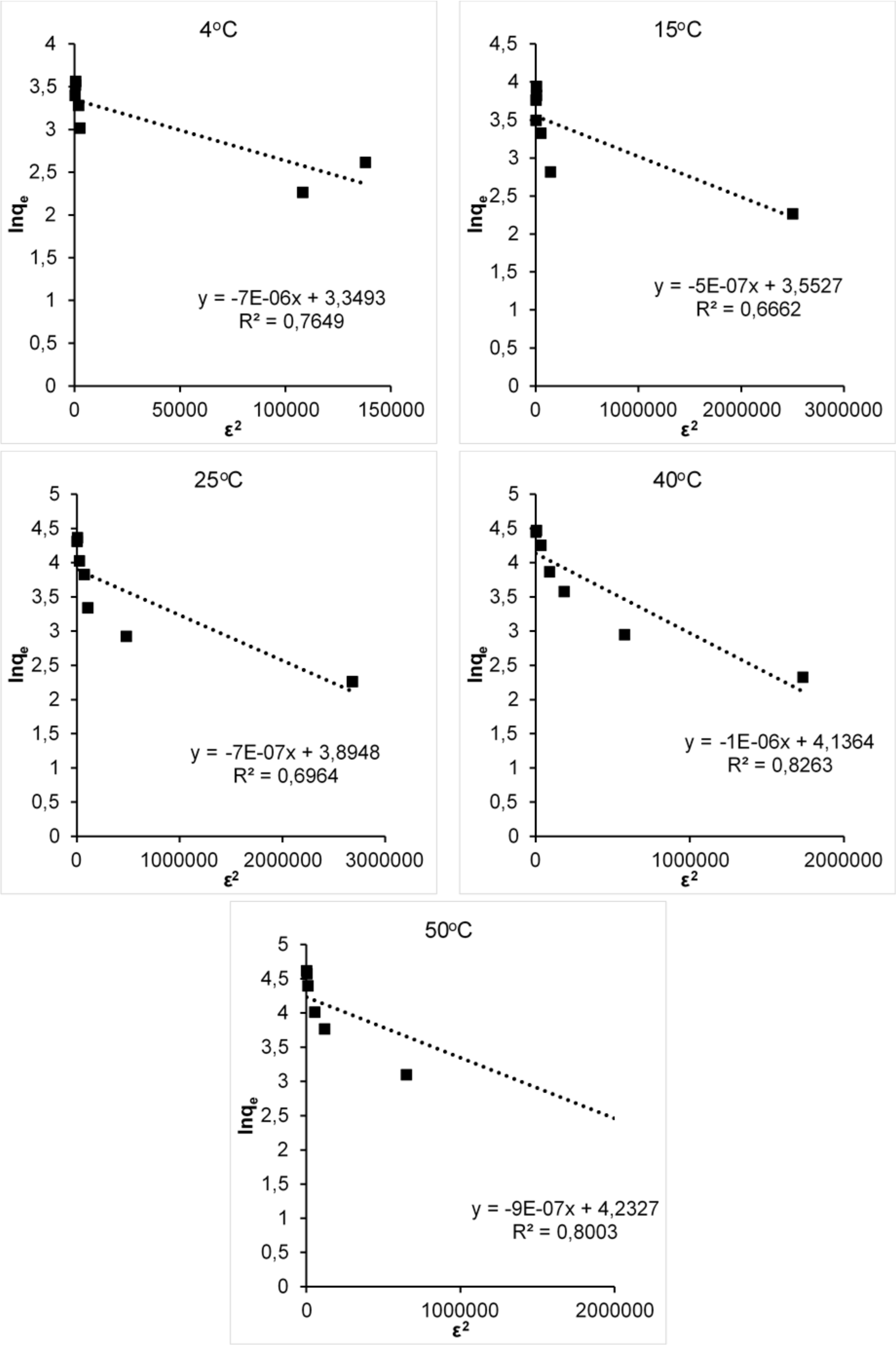

Figure S12. Application of Radushkevich-Dubinin model to adsorption data obtained at different temperatures.

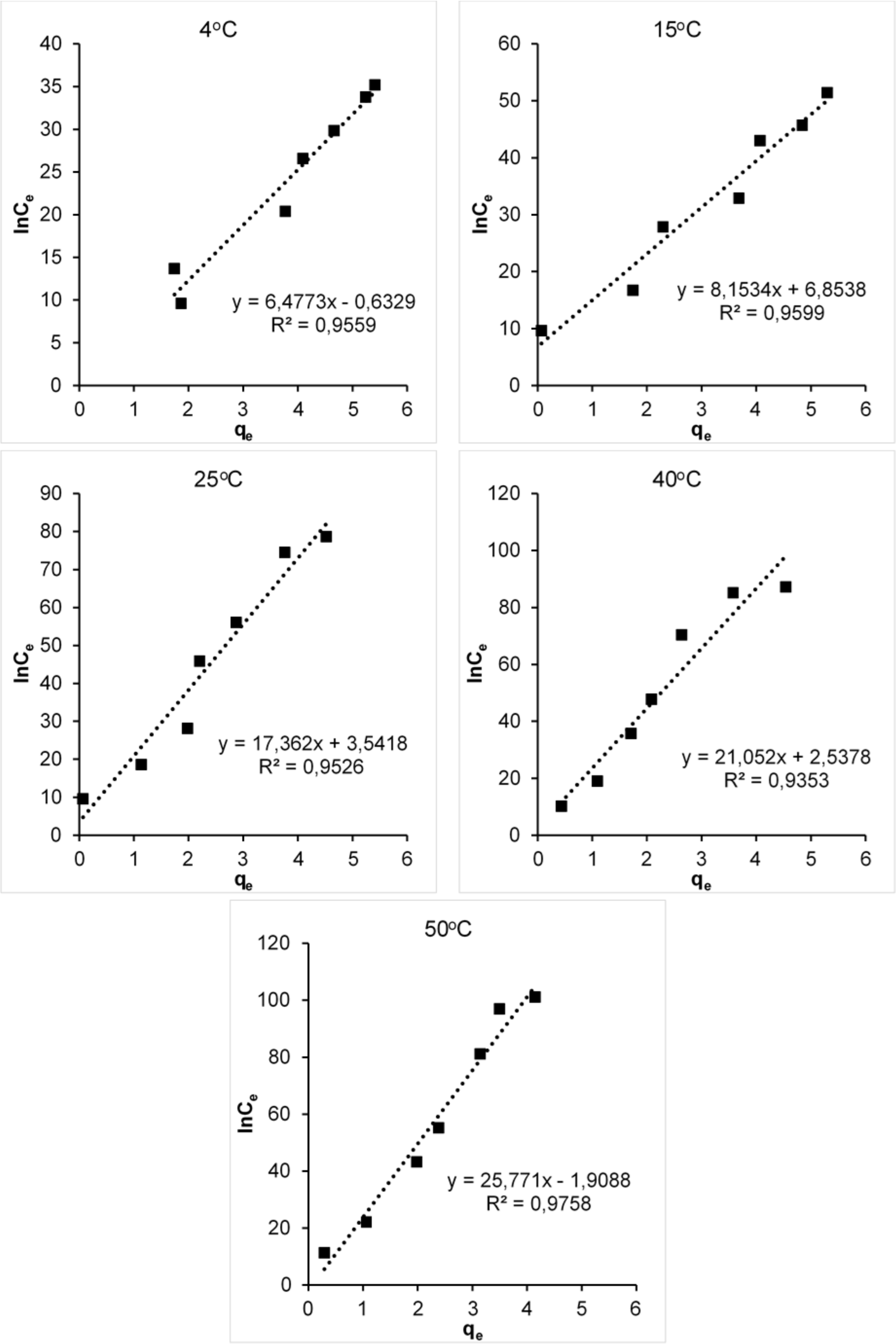

Figure S13. Application of Temkin isotherm model to adsorption data obtained at different temperatures.

Table S7. K_o values changing with temperature.

**Temperature, ˚C**	K_o
4	1.26
15	3.16
25	5.94
40	6.80
50	8.26

References

(1) Zhao, Z.; Fu, D.; Zhang, B. Novel molecularly imprinted polymer prepared by palygorskite as support for selective adsorption of bisphenol A in aqueous solution. *Desalin. Water Treat.* **2016**, *57* (27), 12433-12442.

(2) Wang, H.; Zhang, H.; Jiang, J.-Q.; Ma, X. Adsorption of bisphenol A onto cationic-modified zeolite. *Desalin. Water Treat.* **2016**, *57* (54), 26299-26306.

(3) Sudhakar, P.; Mall, I. D.; Srivastava, V. C. Adsorptive removal of bisphenol-A by rice husk ash and granular activated carbon—A comparative study. *Desalin. Water Treat.* **2016**, *57* (26), 12375-12384.

(4) Genç, N.; Kılıçoğlu, Ö.; Narci, A. O. Removal of Bisphenol A aqueous solution using surfactant-modified natural zeolite: Taguchi's experimental design, adsorption kinetic, equilibrium and thermodynamic study. *Environ. Technol.* **2017**, *38* (4), 424-432.

(5) Qiu, F.; Peng, M.; Wei, Z.; Wang, X.; Yang, J. Preparation of polyethersulfone/sulfonated polyethersulfonephenylethane microspheres and its application for the adsorption of bisphenol A. *J. Appl. Polym. Sci.* **2016**, *133* (9).

(6) Qin, F.-X.; Jia, S.-Y.; Liu, Y.; Li, H.-Y.; Wu, S.-H. Adsorptive removal of bisphenol A from aqueous solution using metal-organic frameworks. *Desalin. Water Treat.* **2015**, *54* (1), 93-102.

(7) Hartono, M. R.; Marks, R. S.; Chen, X.; Kushmaro, A. Hybrid multi-walled carbon nanotubes-alginate-polysulfone beads for adsorption of bisphenol-A from aqueous solution. *Desalin. Water Treat.* **2015**, *54* (4-5), 1167-1183.

(8) Duan, F.; Chen, C.; Zhao, X.; Yang, Y.; Liu, X.; Qin, Y. Water-compatible surface molecularly imprinted polymers with synergy of bi-functional monomers for enhanced selective adsorption of bisphenol A from aqueous solution. *Environ. Sci.: Nano* **2016**, *3* (1), 213-222.

(9) Liu, X.; Hu, Y.; Huang, J.; Wei, C. Detailed characteristics of adsorption of bisphenol A by highly hydrophobic MCM-41 mesoporous molecular sieves. *Res. Chem. Intermed.* **2016**, *42* (9), 7169-7183.

(10) Bayramoğlu, G.; Arıca, M. Y.; Liman, G.; Çelikbiçak, Ö.; Salih, B. Removal of bisphenol A from aqueous medium using molecularly surface imprinted microbeads. *Chemosphere* **2016**, *150*, 275-284.

(11) Salehinia, S.; Ghoreishi, S. M.; Maya, F.; Cerdà, V. Hydrophobic magnetic montmorillonite composite material for the efficient adsorption and microextraction of bisphenol A from water samples. *J. Environ. Chem. Eng.* **2016**, *4* (4), 4062-4071.

(12) Dehghani, M. H.; Ghadermazi, M.; Bhatnagar, A.; Sadighara, P.; Jahed-Khaniki, G.; Heibati, B.; McKay, G. Adsorptive removal of endocrine disrupting bisphenol A from aqueous solution using chitosan. *J. Environ. Chem. Eng.* **2016**, *4* (3), 2647-2655.

(13) Zhang, Z.; Chen, X.; Rao, W.; Chen, H.; Cai, R. Synthesis and properties of magnetic molecularly imprinted polymers based on multiwalled carbon nanotubes for magnetic extraction of bisphenol A from water. *J. Chromatogr. B: Biomed. Sci. Appl.* **2014**, *965*, 190-196.

(14) Liu, Y.; Zhong, G.; Liu, Z.; Meng, M.; Liu, F.; Ni, L. Facile synthesis of novel photoresponsive mesoporous molecularly imprinted polymers for photo-regulated selective separation of bisphenol A. *Chem. Eng. J.* **2016**, *296*, 437-446.

(15) Guo, W.; Hu, W.; Pan, J.; Zhou, H.; Guan, W.; Wang, X.; Dai, J.; Xu, L. Selective adsorption and separation of BPA from aqueous solution using novel molecularly imprinted polymers based on kaolinite/Fe 3 O 4 composites. *Chem. Eng. J.* **2011**, *171* (2), 603-611.

(16) Rathnayake, S. I.; Xi, Y.; Frost, R. L.; Ayoko, G. A. Environmental applications of inorganic–organic clays for recalcitrant organic pollutants removal: Bisphenol A. *J. Colloid Interface Sci.* **2016**, *470*, 183-195.

(17) Dong, R.; Li, J.; Xiong, H.; Lu, W.; Peng, H.; Chen, L. Thermosensitive molecularly imprinted polymers on porous carriers: preparation, characterization and properties as novel adsorbents for bisphenol A. *Talanta* **2014**, *130*, 182-191.

(18) Bele, S.; Samanidou, V.; Deliyanni, E. Effect of the reduction degree of graphene oxide on the adsorption of Bisphenol A. *Chem. Eng. Res. Des.* **2016**, *109*, 573-585.

(19) Zhou, Q.; Wang, Y.; Xiao, J.; Fan, H. Adsorption and removal of bisphenol A, α-naphthol and β-naphthol from aqueous solution by Fe 3 O 4@ polyaniline core–shell nanomaterials. *Synth. Met.* **2016**, *212*, 113-122.

(20) Koduru, J. R.; Lingamdinne, L. P.; Singh, J.; Choo, K.-H. Effective removal of bisphenol A (BPA) from water using a goethite/activated carbon composite. *Process Saf. Environ. Prot.* **2016**, *103*, 87-96.
